# Supplementary material for: Near-ideal spontaneous photon sources in silicon quantum photonics
Source: Nat Commun. 2020 May 19;11:2505. doi: 10.1038/s41467-020-16187-8 (PMC7237445; doi:10.1038/s41467-020-16187-8)
Supplement: Supplementary file 1 — Supplementary Information [file 41467_2020_16187_MOESM1_ESM.pdf]

Near-ideal spontaneous photon sources in silicon quantum photonics

S. Paesani et al.

Supplementary Note 1: Source design details

a. Intermodal four wave mixing

Intermodal four wave mixing occurs when the pump, signal and idler waves propagate on different order modes of a waveguide or fiber. This is different from the usual intramodal four wave mixing, in which all the waves propagate on the same spatial mode. The phase mismatch parameter Δk, making explicit the modal dependence, is written as

$$\Delta k = \frac{\omega_{\mathrm{p1}}}{c} n^{j}_{\mathrm{eff}}(\omega_{\mathrm{p1}}) + \frac{\omega_{\mathrm{p2}}}{c} n^{q}_{\mathrm{eff}}(\omega_{\mathrm{p2}}) - \frac{\omega_{\mathrm{s}}}{c} n^{l}_{\mathrm{eff}}(\omega_{\mathrm{s}}) - \frac{\omega_{\mathrm{i}}}{c} n^{m}_{\mathrm{eff}}(\omega_{\mathrm{i}}), \tag{1}$$

where $\mathrm{p}_{1(2)}$, i, s, refer to the pump, signal and idler respectively and j, q, l, m indicate the order of the waveguide mode excited for each wave. Supplementary Equation (1) considers one pump photon on the j-th order mode, the other pump photon on the q-th order mode, the signal photon on the l-th order mode and the idler on the m-th order mode. By choosing properly the excited modes and by engineering their dispersion profiles, perfect phase matching can be achieved [1]. Peculiar of the intermodal four wave mixing is the discrete band of the phase matching, which enables higher spectral purities without resorting to narrow spectral filtering of the generated photons.

In this work we excited an intermodal combination involving the pump photons on both the TM0 and TM1, and the signal and idler on respectively the TM1 and TM0. We measured the discrete bands of the spontaneous signal and idler at 1588 nm and 1516 nm respectively, as reported in Supplementary Figure 1. The waveguide used has a 2μm $\times$ 0.22μm cross section.

b. Optimal choice of the waveguide length in a pump-delayed excitation scenario

The temporal delay between the pump modes depends on the length of the waveguide L and the difference in their group velocities by:

$$\tau = -\frac{L}{2}\left(\frac{1}{v_0} - \frac{1}{v_1}\right), \tag{2}$$

with v_0 and v_1 the TM0 and TM1 group velocities respectively. This choice ensures that the two pump pulses, associated with the TM0 and TM1 modes, will get maximally overlapped in the middle of the waveguide. The waveguide has to be long enough to accommodate the adiabatic switching of the nonlinear interaction, but at the same time it has to limit the excess loss of signal/idler photons once the pump modes are no more temporally overlapped. In order to estimate the value of L at which this condition starts to occur, we simulated the source joint spectral amplitude (JSA) using the following expression [2]:

$$F(\omega_{\mathrm{s}}, \omega_{\mathrm{i}}) = N \int_0^L dz \int d\omega_{\mathrm{p}}\, \alpha_{\mathrm{p}}\left(\omega_{\mathrm{p}} - \omega^0_{\mathrm{p}}\right)\, \alpha_{\mathrm{p}}\left(\omega_{\mathrm{s}} + \omega_{\mathrm{i}} - \omega_{\mathrm{p}} - \omega^0_{\mathrm{p}}\right) \exp\left(-\mathrm{i}\omega_{\mathrm{p}}\tau\right) \exp\left(-\mathrm{i}\Delta k z\right), \tag{3}$$

where N is a normalisation factor, α_{p} is the pump field envelope, L is the waveguide length, ω^0_{p} is the central frequency of the pump, τ is the temporal delay between the two pumps and Δk is the phase mismatch parameter. Δk is written considering the higher order modes involved in the intermodal process, as in Supplementary Equation (1). In α_{p} is also included the spectral phase of the pump as $\alpha_{\mathrm{p}}(\omega) = A(\omega) \exp\left(\mathrm{i}\, \phi_{\mathrm{p}}(\omega)\right)$, with $A(\omega)$ the field amplitude and $\phi_{\mathrm{p}}(\omega)$ the phase amplitude. From the JSA we calculated the purity of either the signal or idler photons by [3]:

$$P = \int\int d\omega d\omega' |q_{\mathrm{s}}(\omega, \omega')|^2, \tag{4}$$

where $q_{\mathrm{s}}(\omega, \omega') = \int d\omega'' F(\omega'', \omega) F^*(\omega'', \omega')$. The purity as a function of the waveguide length, with the delay set by Supplementary Equation (2), is plotted in Supplementary Figure 2a, using the measured pump amplitude $A(\omega)$ and phase $\phi_{\mathrm{p}}(\omega)$ spectral profiles shown in Supplementary Figure 2b. The longer the waveguide, the more adiabatic is the turn on and off of the nonlinear interaction, and the higher is the purity. However, after a certain length, which corresponds to nearly 7 mm, only a modest improvement of the purity is obtained by further increasing the waveguide length. To better understand this, it is useful to introduce the effective interaction length L_{eff}, defined as [2]:

$$L_{\mathrm{eff}} = \frac{\int_0^L dz \int dt I_{\mathrm{p1}}(z,t) I_{\mathrm{p2}}(z,t)}{\int dt I_{\mathrm{p1}}(z = L/2, t) I_{\mathrm{p2}}(z = L/2, t)}, \tag{5}$$

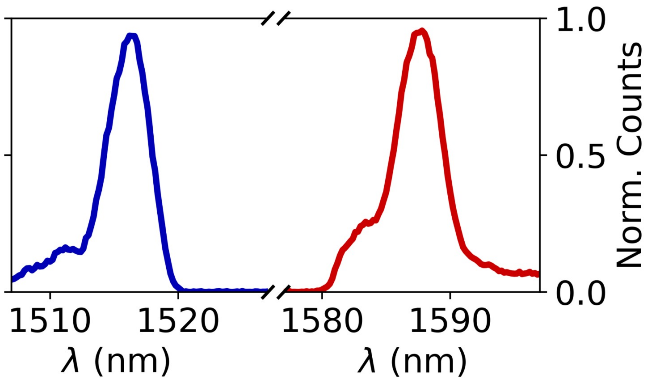

Supplementary Figure 1. Signal and idler experimental spectra from raw single-channel detection events. The measurement was carried out using the same pulsed pump of the experiment.

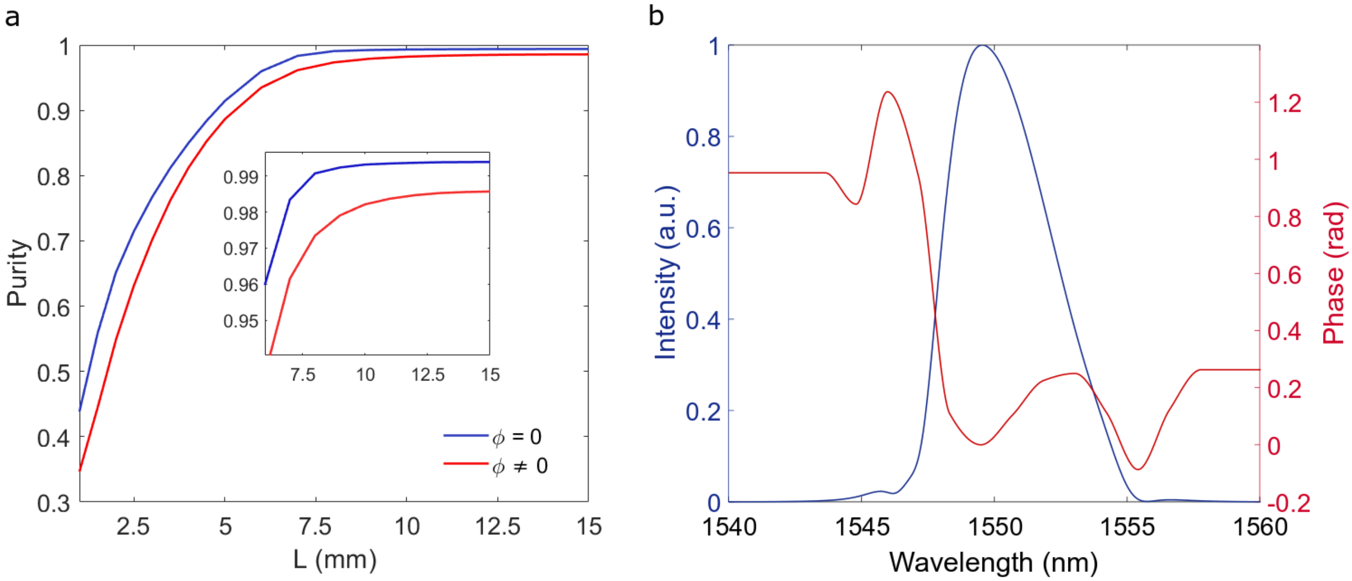

Supplementary Figure 2. a) Simulated purity of the heralded photon state versus the length of the waveguide. In blue the simulation without the effect of the pump phase. In red the simulated purity taking into account the actual pump spectrum employed in our experiment. b) Spectral intensity (blue) and phase (red) of the pump laser light employed in our experiment. The pump intensity and phase reconstruction was performed via a commercial frequency-resolved optical gating (FROG).

where $I_{\rm p1}(z,t)$, $I_{\rm p2}(z,t)$ are the intensities of the two pumps at position z along the waveguide and at time t. The quantity $L_{\rm eff}$ is an indicator of the effective length over which the nonlinear interaction occurs before the two pump pulses lose their spatial overlap inside the waveguide. The optimised delay in Supplementary Equation (2) can be readily demonstrated to maximise the factorisability as well as the effective interaction length $L_{\rm eff}$, thus maximising also the generation efficiency. To be noticed that $L_{\rm eff} < L$ as soon as $v_0 \neq v_1$. $L_{\rm eff}$ has a maximum ($L_{\rm eff}^{\rm max}$) set by the group velocity mismatch and the pulse width, such that for $L > L_{\rm eff}^{\rm max}$ the purity saturates, as shown in Supplementary Figure 2a. Therefore, when designing the device, the length of the multimode waveguide has to be chosen larger than $L_{\rm eff}$. We choose $L = 11\,{\rm mm}$ for our design, such that the purity is saturating above 99% (see Supplementary Figure 2a).

c. JSA engineering through pump-delayed excitation

In the work of Fang et al. [2] it is proposed to use a controlled temporal delay between the two pump pulses of non degenerate SFWM for the generation of spectrally pure single photons. By adequately tuning the delay it is

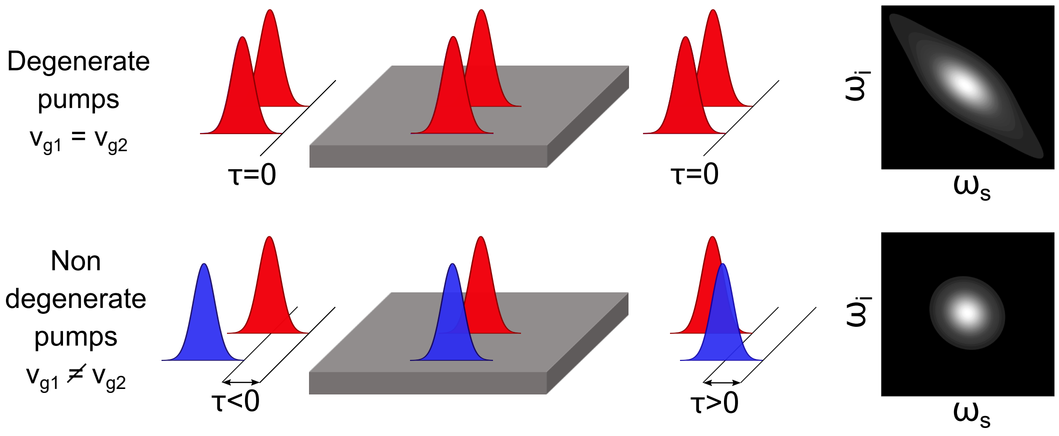

Supplementary Figure 3. Delayed dual pump excitation scenario. (Top figure) When the pumps are degenerate they are injected in the waveguide (gray box) without any temporal delay τ. Therefore, the two pulses travel together before and after the waveguide ($\tau = 0$). In this case, the JSI exhibits low state purity. (Bottom figure) When the pumps are not degenerate, they have different group velocities. With the proper temporal delay ($\tau < 0$), the two pulses will interact only when they are both fully loaded in the sample. Due to the walk-off, at the end of the waveguide the faster pulse is ahead with respect to the slower one ($\tau > 0$). In this case, τ can be chosen in order to have a factorisable biphoton wave function.

possible to shape the phase matching function, giving rise to a factorisable biphoton wavefunction with unit purity. This concept is shown in Supplementary Figure 3.When the pumps are degenerate, i.e. equal in all the degrees of freedom, they have equal group velocities. In this case the pulses are injected in the waveguide (grey box in the figure) with temporal delay $\tau = 0$ and propagate without any walk-off till the end of the device. In this case the joint spectral intensity (JSI) exhibits a reduced state purity. When the pumps are not degeneratethey have different group velocities. By applying the proper temporal delay between the two pulses ($\tau < 0$), they will interact only when they are both loaded in the waveguide. Due to the walk-off, the faster pulse will overtake the slower one, ceasing the interaction before the end of the waveguide. Therefore, at the output of the waveguide the faster pulse will be ahead with respect to the slower one ($\tau > 0$). With the right value of τ, the JSI can be shaped to give almost unit purity.

We exploit the different group velocities of the waveguide modes involved in intermodal FWM. We consider pumps that are degenerate in frequency and with the same pump profile, but that excite different waveguide modes. The joint spectral amplitude (JSA) corresponding to photon pairs emitted by the delayed pump excitation scheme has been given in Supplementary Equation (3); it can be rewritten neglecting the pump phase and assuming a Gaussian pump envelope as [2]

$$F(\omega_\mathrm{s},\omega_\mathrm{i}) = N\int_0^L dz \int d\omega_\mathrm{p} \exp\left[-\left(\frac{\omega_\mathrm{p}-\omega_\mathrm{p}^0}{\sigma}\right)^2\right] \exp\left[-\left(\frac{\omega_\mathrm{s}+\omega_\mathrm{i}-\omega_\mathrm{p}-\omega_\mathrm{p}^0}{\sigma}\right)^2\right] \exp\left[-\mathrm{i}\omega_\mathrm{p}\tau\right]\exp\left[-\mathrm{i}\Delta k z\right], \tag{6}$$

where N is a normalisation factor, L is the waveguide length, ω_p, ω_s, ω_i the pump, signal and idler frequencies, ω_p^0 the central pump frequency, σ is the pump bandwidth, τ is the temporal delay and Δk is the phase mismatch parameter defined in Supplementary Equation (1). By Taylor expanding Δk around the phase matching frequencies and taking only the linear terms, Supplementary Equation (6) can be rewritten as a function of $\Delta\omega_\mathrm{s} = \omega_\mathrm{s} - \omega_\mathrm{s}^0$ and $\Delta\omega_\mathrm{i} = \omega_\mathrm{i} - \omega_\mathrm{i}^0$ [4],

$$F_\mathrm{lin}(\Delta\omega_\mathrm{s},\Delta\omega_\mathrm{i}) = N\alpha\left(\Delta\omega_\mathrm{s},\Delta\omega_\mathrm{i}\right)\phi\left(\Delta\omega_\mathrm{s},\Delta\omega_\mathrm{i}\right), \tag{7}$$

where α and ϕ are given by:

$$\alpha\left(\Delta\omega_\mathrm{s},\Delta\omega_\mathrm{i}\right) = \exp\left[-\frac{\left(\Delta\omega_\mathrm{s}+\Delta\omega_\mathrm{i}\right)^2}{2\sigma^2}\right] \tag{8}$$

$$\begin{aligned}\phi\left(\Delta\omega_\mathrm{s},\Delta\omega_\mathrm{i}\right) &= \exp\left[-\left(\frac{\tau_\mathrm{s}\Delta\omega_\mathrm{s}+\tau_\mathrm{i}\Delta\omega_\mathrm{i}}{\sigma_\mathrm{eff}\tau_\mathrm{p}}\right)^2\right] \\ &\times\left[\mathrm{erf}\left(\frac{\sigma_\mathrm{eff}(\tau+\tau_\mathrm{p})}{2}+\mathrm{i}\frac{\tau_\mathrm{s}\Delta\omega_\mathrm{s}+\tau_\mathrm{i}\Delta\omega_\mathrm{i}}{\sigma_\mathrm{eff}\tau_\mathrm{p}}\right)-\mathrm{erf}\left(\frac{\sigma_\mathrm{eff}\tau}{2}+\mathrm{i}\frac{\tau_\mathrm{s}\Delta\omega_\mathrm{s}+\tau_\mathrm{i}\Delta\omega_\mathrm{i}}{\sigma_\mathrm{eff}\tau_\mathrm{p}}\right)\right],\end{aligned} \tag{9}$$

with $\sigma_{\text{eff}} = \sigma/\sqrt{2}$ the effective bandwidth; the group delays are

$$\tau_{\text{s}} = L\left(\frac{\beta_{1,\text{p1}}(\omega_{\text{p}}^{0}) + \beta_{1,\text{p2}}(\omega_{\text{p}}^{0})}{2} - \beta_{1,\text{s}}(\omega_{\text{s}}^{0})\right), \tag{10}$$

$$\tau_{\text{i}} = L\left(\frac{\beta_{1,\text{p1}}(\omega_{\text{p}}^{0}) + \beta_{1,\text{p2}}(\omega_{\text{p}}^{0})}{2} - \beta_{1,\text{i}}(\omega_{\text{i}}^{0})\right), \tag{11}$$

$$\tau_{\text{p}} = L\left(\beta_{1,\text{p1}}(\omega_{\text{p}}^{0}) - \beta_{1,\text{p2}}(\omega_{\text{p}}^{0})\right), \tag{12}$$

with $\beta_{1,\text{j}}(\omega) = dk_{\text{j}}(\omega)/d\omega$ (j=p,s,i) the inverse of the group velocity for the pump, signal and idler waveguide modes. The phase matching angle results to be [4]

$$\theta_{\text{pm}} = -\arctan\left(\tau_{\text{s}}/\tau_{\text{i}}\right). \tag{13}$$

The factorisability of the biphoton wave function requires the θ_{pm} to be in the range $[0°, 90°]$, therefore the purity condition becomes [2]

$$\tau_{\text{s}}\tau_{\text{i}} \leq 0. \tag{14}$$

Supplementary Equation (14) is satisfied when the idler and signal are anti-symmetric in terms of β_1 with respect to the average β_1 of the pumps, i.e. if $\beta_{1,\text{i}} > \langle\beta_{1,\text{p}}\rangle$ then the $\beta_{1,\text{s}} < \langle\beta_{1,\text{p}}\rangle$, and viceversa, with $\langle\beta_{1,\text{p}}\rangle = \frac{\beta_{1,\text{p1}}+\beta_{1,\text{p2}}}{2}$. This means that the signal and idler group velocities must be respectively greater and lower (and viceversa) than the threshold set by the pump group velocities.

Looking now at the effects of the pump delay, this will affect the effective length L_{eff} of the interaction, given by Supplementary Equation (5), thus conditioning the efficiency and spectral purity of the generated photons. Consider the standard case in which the pumps travel with the same group velocity. Here the interaction occurs, with almost perfect overlap between the pulses, along the entire length of the waveguide. Therefore, the effective length is L and the resulting phase matching function exhibits the usual 'sinc' behaviour as

$$\phi\left(\Delta\omega_{\text{s}}, \Delta\omega_{\text{i}}\right) = \text{sinc}\left(\frac{\tau_{\text{s}}\Delta_{\text{s}} + \tau_{\text{i}}\Delta_{\text{i}}}{2}\right), \tag{15}$$

whose side lobes hinder the generation of unit purity states [5].

Consider now the case of two pump pulses with different group velocities and $\tau = \tau_{\text{p}}/2$, which will be proved to be the delay maximising the state factorisability. The interaction between the two pump pulses starts when they are both already in the sample; the interaction increases adiabatically to the maximum overlap at $z = L/2$, and then decreases and switch off before the faster pulse exits the waveguide. This situation occurs when $|\sigma_{\text{eff}}\tau_{\text{p}}| \gg 1$, which corresponds to long waveguides, large difference between the pump group velocities, or short pulse duration of the pumps [2]. The interaction length in this case becomes

$$L_{\text{eff}} = \frac{\sqrt{2}}{|\sigma_{\text{eff}}\tau_{\text{p}}|}L, \tag{16}$$

which is a small value compared to L when the optimal delay is used. Despite the reduced interaction length and the lower efficiency, the phase matching function now exhibits a Gaussian profile,

$$\phi\left(\Delta\omega_{\text{s}}, \Delta\omega_{\text{i}}\right) = \exp\left[-\left(\frac{\tau_{\text{s}}\Delta\omega_{\text{s}} + \tau_{\text{i}}\Delta\omega_{\text{i}}}{\sigma_{\text{eff}}\tau_{\text{p}}}\right)^2\right]. \tag{17}$$

At this point, the JSA in Supplementary Equation (7) can be rewritten using the pulse envelope in Supplementary Equation (8) and the phase matching function in Supplementary Equation (17), becoming

$$F_{\text{lin}}\left(\Delta\omega_{\text{s}}, \Delta\omega_{\text{i}}\right) \approx \exp\left[-\frac{\left(\Delta\omega_{\text{s}} + \Delta\omega_{\text{i}}\right)^2}{2\sigma^2}\right]\exp\left[-\left(\frac{\tau_{\text{s}}\Delta\omega_{\text{s}} + \tau_{\text{i}}\Delta\omega_{\text{i}}}{\sigma_{\text{eff}}\tau_{\text{p}}}\right)^2\right], \tag{18}$$

which exhibits a Gaussian profile, being the product of two Gaussian functions. By using Supplementary Equation (18) and Supplementary Equation (4), an analytical expression for the purity P can be found [2],

$$P = \sqrt{\frac{\left(\frac{\tau_{\rm i}}{\tau_{\rm p}} - \frac{\tau_{\rm s}}{\tau_{\rm p}}\right)^2}{\left(\frac{1}{2} + 2\left(\frac{\tau_{\rm s}}{\tau_{\rm p}}\right)^2\right)\left(\frac{1}{2} + 2\left(\frac{\tau_{\rm i}}{\tau_{\rm p}}\right)^2\right)}}. \tag{19}$$

By solving Supplementary Equation (19) for $P = 1$ the following relation is found,

$$\tau_{\rm s}\tau_{\rm i} + \frac{\tau_{\rm p}^2}{4} = 0, \tag{20}$$

which gives the conditions for perfect factorisability of the JSA.

From here on, we will neglect the frequency dependence of the β_1's, such that $\beta_{1,\rm j}\left(\omega_{\rm j}^0\right) \equiv \beta_{1,\rm j}$, (j=p1,p2,s,i). By considering the average β_1 of the pumps

$$\langle\beta_{1,\rm p}\rangle = \frac{\beta_{1,\rm p1} + \beta_{1,\rm p2}}{2}, \tag{21}$$

and the following relation

$$\beta_{1,\rm p1} - \beta_{1,\rm p2} = 2\left(\langle\beta_{1,\rm p}\rangle - \beta_{1,\rm p2}\right) = 2\left(\beta_{1,\rm p1} - \langle\beta_{1,\rm p}\rangle\right), \tag{22}$$

$\tau_{\rm p}^2$ and $4\tau_{\rm s}\tau_{\rm i}$ can be rewritten as

$$\tau_{\rm p}^2 = 4L^2\left(\langle\beta_{1,\rm p}\rangle - \beta_{1,\rm p2}\right)\left(\beta_{1,\rm p1} - \langle\beta_{1,\rm p}\rangle\right), \tag{23}$$

and

$$4\tau_{\rm s}\tau_{\rm i} = 4L^2\left(\langle\beta_{1,\rm p}\rangle - \beta_{1,\rm i}\right)\left(\langle\beta_{1,\rm p}\rangle - \beta_{1,\rm s}\right). \tag{24}$$

Therefore, from Supplementary Equation (23), Supplementary Equation (24) and Supplementary Equation (20), it follows

$$\left(\langle\beta_{1,\rm p}\rangle - \beta_{1,\rm p2}\right)\left(\beta_{1,\rm p1} - \langle\beta_{1,\rm p}\rangle\right) = \left(\langle\beta_{1,\rm p}\rangle - \beta_{1,\rm i}\right)\left(\beta_{1,\rm s} - \langle\beta_{1,\rm p}\rangle\right), \tag{25}$$

which is satisfied when

$$\beta_{1,\rm p1} = \beta_{1,\rm s}, \tag{26}$$

$$\beta_{1,\rm p2} = \beta_{1,\rm i}. \tag{27}$$

or

$$\beta_{1,\rm p1} = \beta_{1,\rm i}, \tag{28}$$

$$\beta_{1,\rm p2} = \beta_{1,\rm s}. \tag{29}$$

The relations in Supplementary Equation (26) and Supplementary Equation (27) and those in Supplementary Equation (28) and (29) set a constraint between the group velocities of the waves involved in the four wave mixing process, stating that unit purity is achieved when the idler has the same group velocity of the faster pump and the signal the same of the slower pump, or vice versa. This condition for factorisability can be obtained with intermodal four wave mixing when the first pump has the same mode of the idler and the other pump has the same mode of the signal, or vice versa.

In Supplementary Figure 4 we report the simulated β_1 for the TM0 and TM1 modes is for the waveguide used in our experiment, with 2μm $\times$ 0.22μm cross section. Supplementary Equation (26) and Supplementary Equation (27) are almost satisfied, with the β_1's which differ by less than 0.3%. In fact, the phase matching wavelengths (arrows in figure) are slightly different from the wavelengths perfectly satisfying Supplementary Equation (26) and Supplementary Equation (27) (dots in figure).

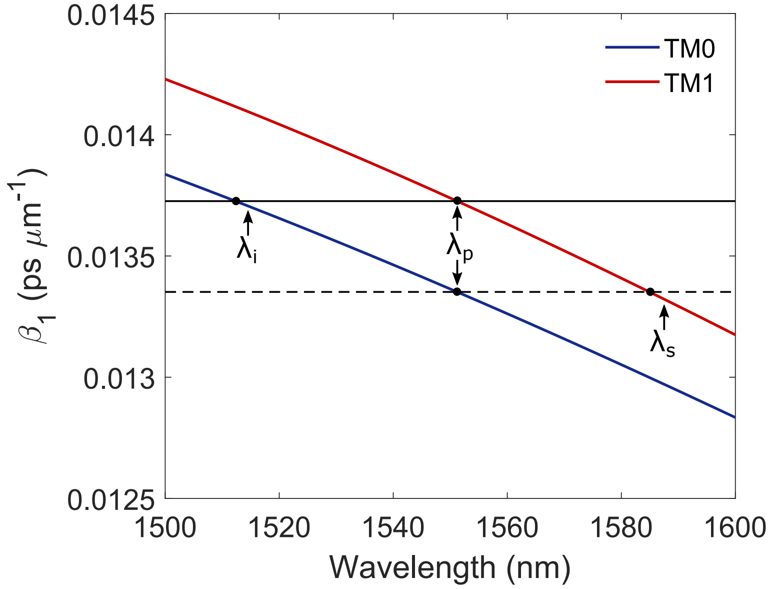

Supplementary Figure 4. Simulated β_1 for TM0 and TM1 in a 2μm $\times$ 0.22μm waveguide. The black lines highlight the $\beta_{1,\mathrm{p1}}$ (dashed) and $\beta_{1,\mathrm{p2}}$ (solid). The black arrows indicate the phase matching wavelengths. The black dots are the wavelengths satisfying Supplementary Equation (26) and Supplementary Equation (27). A slight mismatch between arrows and dots is present, suggesting that Supplementary Equation (26) and Supplementary Equation (27) are not perfectly satisfied at the phase matching wavelengths.

d. Spiral multi-modal source design and modal crosstalk in Euler bends

A crucial step in the design of the compact spiral source is the choice of the bend. Indeed, it is well known that different mode orders of a multimode straight waveguide are scrambled in a tight bend. Ideally, the two pump modes, and the ones of the signal and the idler, should be only coupled by the non-linearity of the material, and any linear interaction which lead to power transfer among modes must be avoided. The detrimental effect would be twofold: on one side, part of the pump power will be converted to mode orders which are out of phase matching, and on the other hand it would prevent the coherent build-up of the signal and idler power along the waveguide. In both cases, the amount of squeezing will be lower, and the shape of the JSA will depart from the one predicted due to the variable phases acquired by the modes after each scrambling event. In order to reduce the modal cross-talk, and at the same time to keep a small foot-print, Euler bends are implemented. These have a curvature $\rho = \frac{1}{R(s)}$ which linearly changes along the bend coordinate s, from a minimum value of zero to a maximum of $\frac{1}{R_{\mathrm{min}}}$, where R_{min} is called the minimum bending radius. A 90° bend has a length of $L = R_{\mathrm{min}}\pi$, and the local bending radius for $s \in \{0, R_{\mathrm{min}}\frac{\pi}{2}\}$ is given by $R(s) = \frac{\pi R_{\mathrm{min}}^2}{2s}$. The bend can be equivalently described by its effective radius R_{eff}, that for a 90° bend is related to R_{min} by $R_{\mathrm{eff}} = 1.87R_{\mathrm{min}}$ [6]. The effective radius is equal to the one of the 90° circular arc that joins the same starting and ending points of the Euler curve. In order to evaluate the bending induced modal crosstalk on a specific straight waveguide mode $|E_{\mathrm{in}}\rangle = |TM_{\mathrm{in,straight}}\rangle$, we divided the bend length in $N = 60$ steps of length ds, and the field propagation between the steps is accomplished through eigenmode expansion. The value of ds has been chosen sufficiently small to consider the local curvature $\rho(s)$ as constant within each step. The method expands the field $|E_{\mathrm{k}}\rangle$ at the input of step k on the local transverse mode basis $\left|TM^{(\mathrm{k})}\right\rangle$, and relates this field to the one at the input of the next step as $|E_{\mathrm{k+1}}\rangle = M^{(\mathrm{k+1})}P^{(\mathrm{k})}M^{(\mathrm{k})}\,|E_{\mathrm{k}}\rangle$, where $M^{(\mathrm{k})} = \sum_{\mathrm{q}} \left|TM_{\mathrm{q}}^{(\mathrm{k})}\right\rangle \left\langle TM_{\mathrm{q}}^{(\mathrm{k})}\right|$ is the projector operator on the local basis and $P^{(\mathrm{k})} = \sum_{\mathrm{q}} \mathrm{e}^{\mathrm{i}\beta_{\mathrm{q}}^{(\mathrm{k})}ds} \left|TM_{\mathrm{q}}^{(\mathrm{k})}\right\rangle \left\langle TM_{\mathrm{q}}^{(\mathrm{k})}\right|$ is the local eigenmode propagation operator. Within each step, the modes are assumed to freely propagate with local wavevectors $\beta_{\mathrm{q}}^{(\mathrm{k})} = \frac{2\pi n_{\mathrm{eff,q}}^{(\mathrm{k})}}{\lambda}$, where $n_{\mathrm{eff,q}}^{(\mathrm{k})}$ are the effective indices of a bent waveguide with radius of curvature $R^{(\mathrm{k})} = \frac{1}{\rho(s_{\mathrm{k}}=k\cdot ds)}$. The projection operators and the modal propagation constants are calculated using the Mode Expansion module of Lumerical FDTD [7]. The crosstalk $|S_{\mathrm{in,q}}|^2$ between the input mode $|TM_{\mathrm{in,straight}}\rangle$ and the straight mode q at the output of the bend is given by $|S_{\mathrm{in,q}}|^2 = |\,\langle TM_{\mathrm{q,straight}}|\, M^{(\mathrm{tot})}\,|TM_{\mathrm{in,straight}}\rangle\,|^2$, where $M^{(\mathrm{tot})} = \prod_{\mathrm{k=1}}^{\mathrm{N-1}} M^{(\mathrm{k+1})}P^{(\mathrm{k})}M^{(\mathrm{k})}$ is the propagation operator

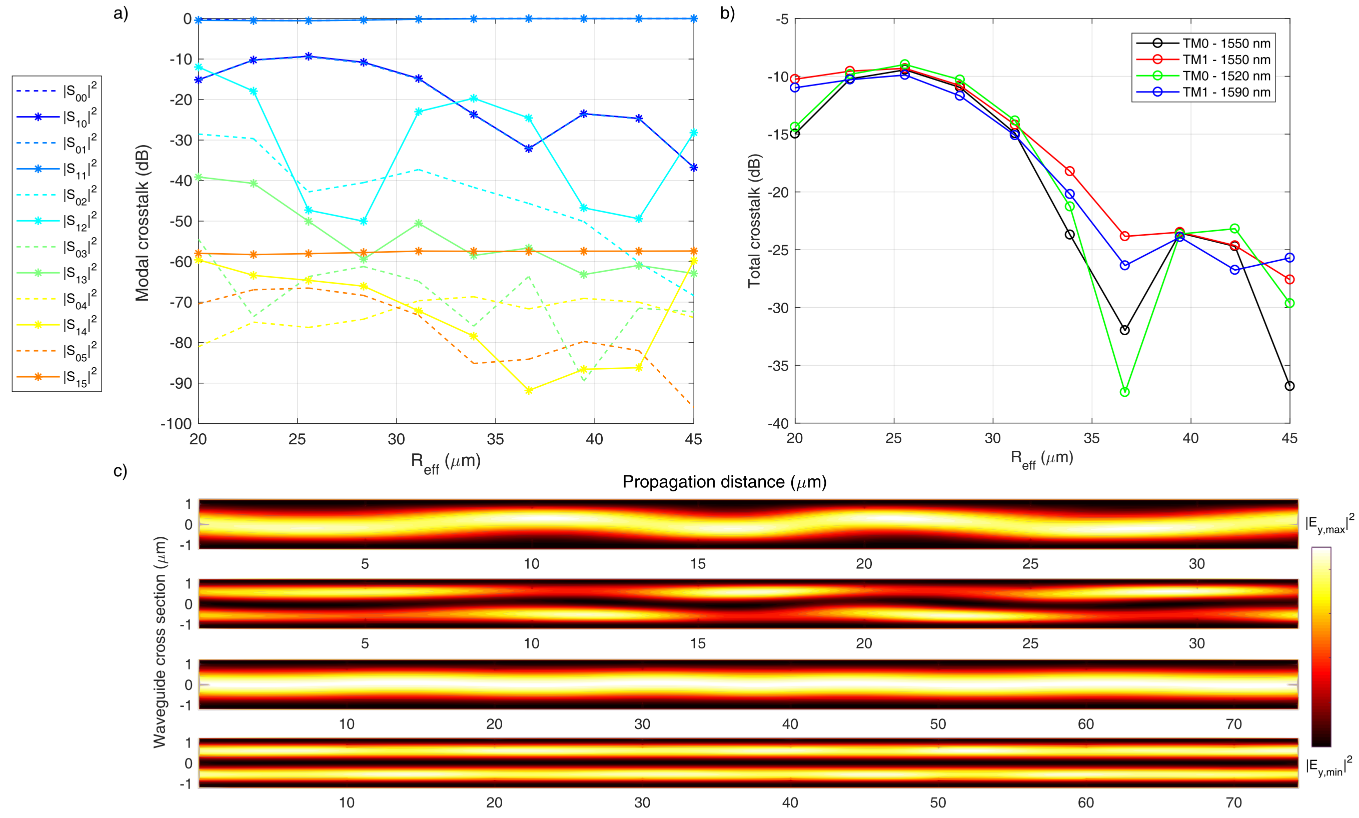

Supplementary Figure 5. (a) Scattering matrix coefficients $|S_{\mathrm{xy}}|^2$ describing the power coupling between mode $\mathrm{TM_x}$ and mode $\mathrm{TM_y}$ when two straight waveguide sections are connected by a 90° Euler bend of effective radius R_{eff}. In this simulation, the wavelength is set to 1550 nm. (b) Total power cross-talk from mode $\mathrm{TM_x}$ at the input of the 90° Euler bend to all other mode orders $\mathrm{TM}_{\mathrm{y}\neq\mathrm{x}}$ at its output, given by $\sum_{\mathrm{y}\neq\mathrm{x}}|S_{\mathrm{y}}|^2$. (c) Electric field intensity distribution in the transverse plane of the waveguide along the direction of propagation the bend, evaluated at different bending radii and mode orders. From the top to the bottom panel: TM0 and $R_{\mathrm{eff}} = 20\,\mu$m, TM1 and $R_{\mathrm{eff}} = 20\,\mu$m, TM0 and $R_{\mathrm{eff}} = 45\,\mu$m and TM1 with $R_{\mathrm{eff}} = 45\,\mu$m.

in the Euler bend. In Supplementary Figure 5(a), we show the the modal crosstalk of the pump modes TM0 and TM1 at 1550 nm as a function of R_{eff}. In both cases, mode conversion mainly occurs among the two lowest order modes, while the crosstalk with the TM2 or higher order modes is below -20 dB. In Supplementary Figure 5(b) we report the total crosstalk, defined as $\sum_{q\neq\mathrm{in}}|S_{\mathrm{in,q}}|^2$, also for the Signal photon at 1520 nm on TM0 and for the Idler photon at 1590 nm on TM1. We see that, in order to keep the total crosstalk below a safe margin of -20 dBm and for all the photons, the effective radius should be higher than 35 μm. To further relax this condition, we choose 45 μm for our design, which ensures a total crosstalk level lower than -25 dB. The evolution of the TM0 and TM1 mode profiles along the bend with $R_{\mathrm{eff}} = 45\,\mu$m is shown in the two lower panels of Supplementary Figure 5(c), where we can notice the absence of beatings due to the very low level of mode mixing. The analysis of the field distribution helps to understand why the level of total crosstalk decreases for $R_{\mathrm{eff}} < 25\,\mu$m, which may suggest to investigate lower bending radius for our design. As it can be noticed from the two upper panels of Supplementary Figure 5(c), which are relative to a bend of $R_{\mathrm{eff}} = 20\,\mu$m, this results from a self-imaging effect, analogous to what happens in a multi-mode interferometer (MMI) devices. Even if this operating condition benefits of a footprint reduction, it is not suited for our purposes, since the coherence between the modes has to be maintained for all the length of the waveguide, hence even in bends, where a significant part of the nonlinear interaction occurs.

Supplementary Note 2: Device and set-up characterisation

a. Characterisation of channel losses and detection efficiencies.

We here report the system efficiencies for the components used in our experimental set-up and integrated circuit (see Supplementary Figure 6). This characterisation was also used to estimate the transmission of the channel for the

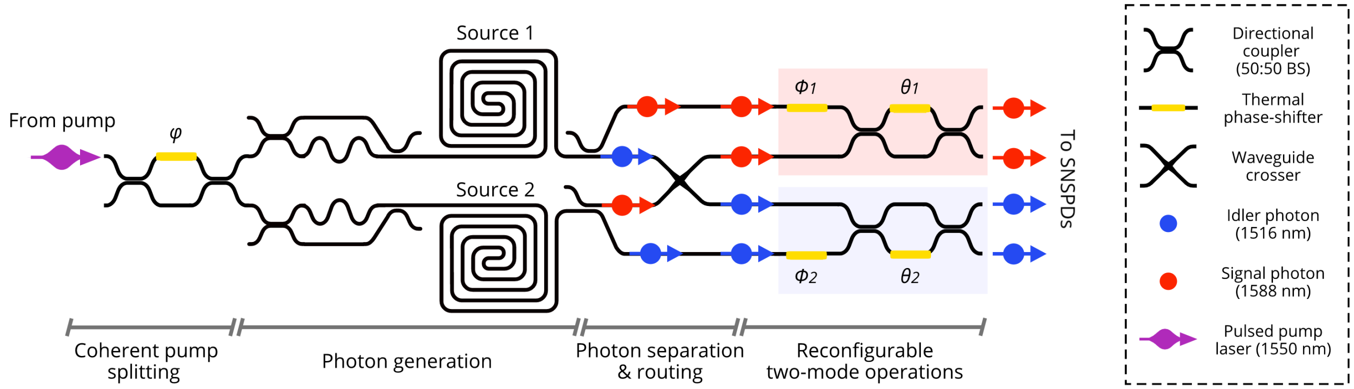

Supplementary Figure 6. Enlarged detailed schematic of the integrated circuit used to perform multi-source interference experiments. Adjustable coherent pump between the two sources is obtained by reconfiguring the MZI φ at the input. After photons are generated in the two multi-modal sources, the output mode-converter deterministically separates idler and signal photons, which are then grouped via the use of a waveguide crossing. Arbitrary and reconfigurable two-mode unitary operations of the form $U(\theta, \phi) = R_Y(\theta)R_Z(\phi)$, with R_Y and R_Z arbitrary rotations along the Y and Z axis of the Bloch sphere respectively, are then performed on the signal (idler) modes via the phase ϕ_1 (ϕ_2) and the MZI θ_1 (θ_2).

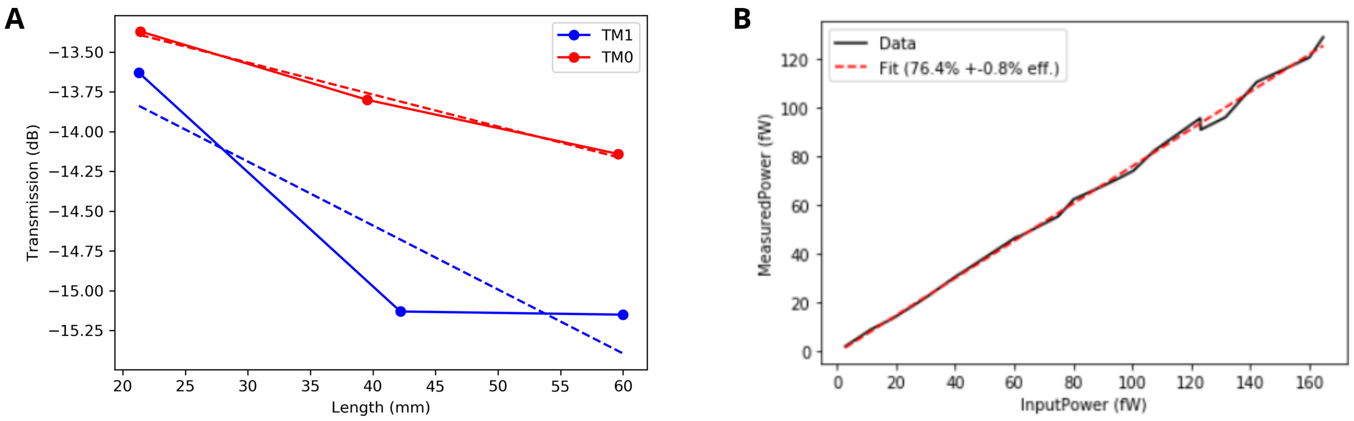

Supplementary Figure 7. **a)** Characterisation of the linear propagation losses in the multimode waveguide employed in the source via cut-back methods. Data are taken on test structures with multimode waveguides of different lengths. Red and blue data are for TM0 and TM1 modes, respectively, and dashed lines are linear fits. The transmission losses estimations from the fits are −0.19 dB/cm for TM0 and −0.40 dB/cm for TM1. **b)** Characterisation of the detection efficiency for the SNSPD employed in the heralding efficiency measurements. Input power correspond to the characterised input power of an attenuated pulsed laser, while the measured power corresponds to the power associated to the single photon counts obtained from the detector. Dark counts in the detection were approximately 100 Hz, corresponding to a signal-to-noise ratio > 1000 in typical measurement conditions.

heralded signal photon to the detectors in the intrinsic heralding efficiency estimation of our source design. Cut-back methods were used to estimate transmission losses in the multi-mode waveguide employed in the sources. Results for both TM0 and TM1 transmission at different waveguide lengths are reported in Supplementary Figure 7a. The loss estimates from the fits are −0.19 dB/cm and −0.40 dB/cm, respectively. The TM grating couplers used in the pump, signal, and idler modes were designed to have optimal efficiency at their respective wavelengths. The off-chip fibre coupling losses were estimated for each type of grating couplers by testing the insertion loss over a set of 15 test structures consisting of a pair of TM grating couplers and a short (approximately 100 μm long) waveguide connector. The grating coupler losses for the signal and idler wavelengths were −6.5(5) dB and −6.6(5) dB, respectively, where uncertainties indicate one standard deviation in the measured samples. The transmission of the off-chip channel to the detector, which included fibre connectors and the pump-rejection filter, was characterised using classical CW laser light at the signal wavelength, obtaining a transmission of −0.99(2) dB. The uncertainty is here given by the measured intensity fluctuations through the channel when spanning the signal photon bandwidth via tuning the CW

		Input	
		TM0	TM1
Output	TM0	-12.67 dB	-43.08 dB
	TM1	-43.18 dB	-12.64 dB

Supplementary Table 1. Characterisation of the transmission in a test mode-converter structure for different input and output transverse modes. For both mode combinations, an extinction higher than 30 dB is measured between the TM0 and TM1 modes.

laser wavelength. The detection system efficiency characterisation for the SNSPD used in the heralding efficiency experiment is reported in Supplementary Figure 7b. The characterisation was performed in the same experimental conditions as for the heralding efficiency experiment. A linear fit of the measured power as a function of the injected one provides an estimate for the system detection efficiency of 76.4(8)%. The overall characterised transmission of the channel of the signal photon from the source in Supplementary Figure 1c, consisting of the TM grating coupler, the off-chip fibre channel, and the detector, is then estimated by combining the characterised efficiencies of these components, obtaining a channel transmission of 14(1)%. This value was used to estimate the intrinsic heralding efficiency of the source via correcting the external losses affecting the signal photon, as discussed in the main text.

b. Mode converters and directional couplers design and characterisation

An important element in the source design is the mode converter for multiplexing and de-multiplexing the TM0 and TM1 modes in and out of the MM source waveguide. To operate the sources correctly, it is important to avoid any modal cross-talk in the mode-converter. Characterisation of the mode-converters designed for our source is reported in Supplementary Table 1. For both TM0 and TM1 the modal cross-talk is measured to be below -30 dB extinction. Regarding the insertion losses of the couplers, full 3D finite element simulations of the 50:50 directional coupler and mode converter provide an insertion loss estimation of < 0.01 dB and 0.1 dB, respectively.

c. TM waveguide crossings design and characterisation

The design of the TM crossings employed in the circuit in Fig. 2a is based on the modal self-imaging technique (see e.g., Ref. [8]). Starting from the design considerations reported in Ref. [9], we adopted a full 3D FDTD simulation to optimize the crossing performance. Simulations indicate an insertion loss of 0.3 dB at a wavelength of 1520 nm, 0.4 dB at 1550 nm and 0.8 dB at 1580 nm. Cross-talk level is kept below -35 dB over the whole spectral interval under consideration. Experimental characterisation of the crossings using the cut-back method provide results consistent with the simulations: measured losses are 0.3(4) dB/cross at 1520 nm, 0.4(4) dB/cross at 1550 nm and 0.4(5) dB/cross at 1580 nm.

Supplementary Note 3: Source performance and linear optical quantum computing fault-tolerance requirements

a. Performance comparison with state-of-the-art sources

For completeness, we here discuss how our sources improves on previous state-of-the-art integrated photon sources reported. As a first example, we simulated the waveguide geometry of Ref.[10], which is a single mode spiral source of 220 nm height, 0.5 μm width and 1.4 cm length. Here, it is necessary to apply a spectral filter to raise the single photon purity to 99%, i.e., the same level of our work, which results in a filtering heralding efficiency of only 14%. To this end, we considered ideal flat filters of 3.5 nm bandwidth, centred at 1565 nm and 1537 nm, and the same pump spectra of our experiment. By considering a typical value of propagation loss of 3 dB cm^{-1}, the heralding efficiency drops to 5.7%. In a standard ring resonator geometry, where no engineering of the coupling section is implemented and no spectral filters are applied, as in Ref.[11], the purity is fundamentally upper limited to 93% [3]. Typically, the gap between the bus and the ring is adjusted to set the resonator in critical coupling operation, because this condition maximises the brightness of the source. As a side effect, the heralding efficiency drops to 50% [12]. We note, however, that promising proposals for improving these limitations of the standard ring designs have recently emerged, which use wavelength-selective coupling to the ring [3] or engineering of the pump spectrum [13]. Using the former approach, a ring source in silicon with 95% purity and 52% efficiency have very recently been reported [14].

We also remark that, while ring resonators have improved compactness and brightness, they require active resonance tuning in order to achieve indistinguishability [15]. In contrast, our sources have the desirable property of being fully passive devices, achieving indistinguishability without the need of any active tuning. In the work of Spring *et. al.* [16], that we consider as a benchmark for waveguides geometries, a nearly pure emission (87%) is obtained by tailoring the group velocity dispersion of the two transverse polarisations. The purity is further raised to 97% through the use of wide-band spectral filters, which produces a final filtering efficiency of 92%. Waveguides have a propagation loss of 0.4 dB/cm and a length of 2.3 cm, that if lumped at the end of the waveguide, give as an upper limit to the heralding efficiency of 75%. Furthermore these sources suffer from fluorescence noise in the silica waveguides used. In the micro-resonator geometry of the work of Lu *et. al.* [17], the maximum preparation efficiency, at the net of detectors, is 82%, and is obtained by strongly over-coupling the resonator to the bus waveguide. By correcting for the detector timing jitter, the estimated purity is about 90%.

When operated in the continuous-wave (CW) pumping regime, ring resonators sources achieve significantly higher brightness compared to waveguide sources, in addition to miniaturisation advantages [18]. However, the energy conservation imply strong frequency anti-correlation between the signal and the idler photons when pumping with CW light. Moreover, as in a CW pumping regime photon pairs are emitted at random times, the probability that two or more pairs are simultaneously emitted is typically negligible. Therefore, with any source design, a CW pumping configuration is ineffective for multi-photon experiments with uncorrelated photons, which is the main aim of this work. Nevertheless, CW-pumped ring sources can be good candidates for two-photon applications where high-rates are required and spectral separability is not needed, such as entanglement-based quantum key distribution protocols (see, e.g., applications in Ref. [10]).

Overall, when considering the logical errors induced by the source noises, our sources represent an order of magnitude improvement respect to the state-of-the art, which is a decisive improvement towards devices able to support scalable photonic quantum information processing, and ultimately fault-tolerance. The high heralding efficiency achieved is highly relevant to multiplexing schemes, where the heralding rate exponentially scales with the number of simultaneously heralded photons [19], and to scattershot-type approaches to sampling algorithms [20].

b. Integration with on-chip mode-locked pump lasers

Important steps have been made in the last few years in the field of integrated mode-locked lasers. A good candidate technology for the integration of the pump laser into our chip is the hetero-integration of electrically pumped III-V materials on a SOI wafer which hosts passive, low-loss components. For example, in Ref. [21] Davenport *et al.* have recently reported the hetero-integration of a mode locked laser with 20 GHz repetition rate, 98 mW of peak power and a sech^2 shaped pulse width of 900 fs, which are all values very close to the ones used in our experiment (except the repetition rate, which is much better in the on-chip laser). The monolithic interconnection with such devices can provide a viable solution to feed the pump laser to our sources directly on-chip, and improve the scaling towards large-scale devices.

c. Improving the source performance towards photonic fault-tolerance requirements

Fault-tolerance in linear-optical quantum computing architectures as the one reported in Ref. [22] require single photons with high purity and indistinguishability to achieve logical error rates at the threshold for error-correction. An analysis on how imperfections in the photonic internal degrees of freedom, resulting in a pair-wise HOM visibility $\mathcal{V} \leq 1$, affect error rates on the large physical lattices obtained in these architectures was performed in Ref. [23]. A lower bound on the local error rates is given by $e \simeq 14(1-\mathcal{V})$. To achieve error rates of approximately 1%, compatible with surface codes error-correction thresholds [24], this implies HOM visibilities of $\gtrsim 99.9\%$. To reach such a high visibility with photons emitted from different sources, both the purity and the indistinguishability need to be higher than 99.9% as well [25].

The indistinguishability is mainly affected by the fabrication tolerance of the device. In Supplementary Figure 8a we simulated the overlap between the JSAs of two sources: one fabricated with the nominal geometrical parameters ($2 \times 0.220\,\mu\text{m}^2$) and the other one with the same height but the width varying by $\pm\,10$nm. The simulation shows that within a width deviation range of about ± 4 nm the sources are indistinguishable by more than 99.9%. The simulation has been performed with the same pump and experimental parameters (pump delay, filters) of the experiment in the main text.

Regarding the purity, the pump properties play a crucial role. We simulated in Supplementary Figure 8b the JSI with the same waveguide cross section and length of the experiment ($2 \times 0.220\,\mu\text{m}^2$, L = 11 mm) but with optimised pump spectrum and phase. We used a perfect Gaussian profile instead of the quasi-Gaussian envelope of

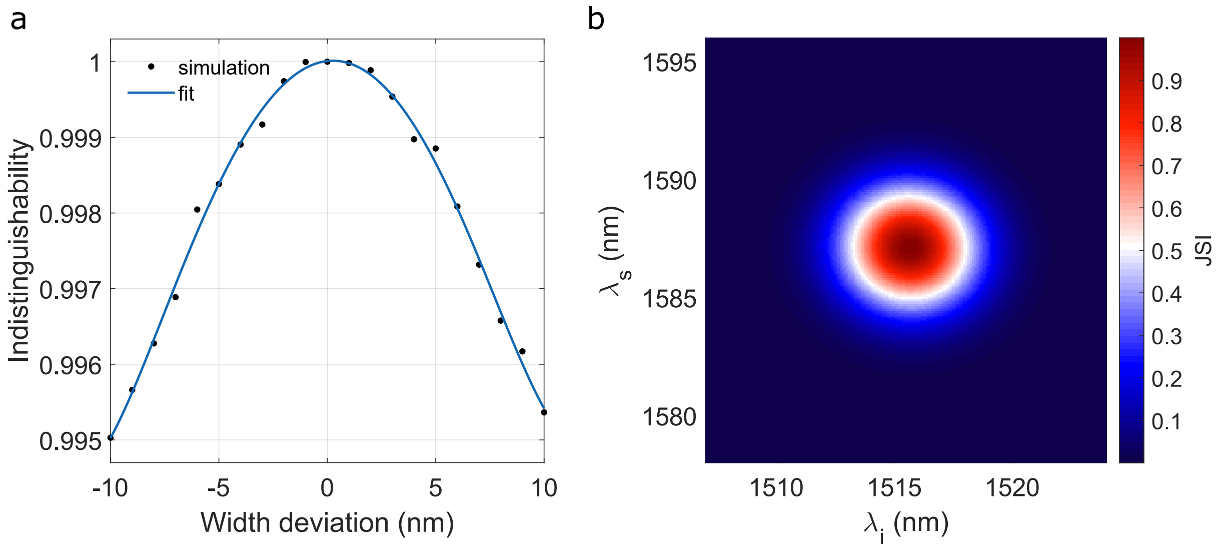

Supplementary Figure 8. a) Overlap integral between the JSAs of two sources versus the width variation of one of the sources. The black dots are the simulated indistinguishabilities. The blue line is a polynomial fit. b) JSI with optimised pump spectrum and phase.

the experiment (see Supplementary Figure 2b) and we considered the phase to be zero. The bandwidth used for the simulation was 4.58 nm, as in the experiment. Without any spectral filtering, the resulting purity is 99.95%. Even larger purities can be achieved with longer waveguides, as already investigated in Supplementary Note 1. As an example, with L = 20 mm and the optimised pump, the purity is 99.97%.

This analysis suggests that her-HOM visibilities greater than 99.9% can be readily achieved with minor improvements using our approach.

Supplementary Note 4: Estimated performance in photonic NISQ architectures

a. Rate estimations for scattershot boson sampling experiments

We here investigate the potential of our sources for near-term noisy intermediate scale quantum (NISQ) photonic architectures by investigating the expected count rates when scaling up the component integration. In particular, we focus on scattershot boson sampling (SBS), which is an architecture for near-term photonic devices for sampling algorithms particularly suited for spontaneous photon sources [20]. In SBS, $n/2$ pairs of photons (i.e. n photons in total) are emitted from a large array of $k > n$ integrated spontaneous photon pair sources. The $n/2$ idler photons are detected to herald the position of the $n/2$ signal photons (which is random at every round of the algorithm), and the signal photons are then injected in a m mode interferometer. The rate for events with n total photons (which include both idler and signal photons) is given by [20]:

$$R(n,k,m) = R_0 \left[\binom{k}{n/2} \tanh(\xi)^n \operatorname{sech}(\xi)^{2k} \right] \eta_{\mathrm{u}}^{mn/2} \eta^n, \tag{30}$$

where R_0 represents the repetition rate of the pump, ξ the squeezing of the spontaneous sources (assumed for simplicity to be uniform across all the sources array). The factor $\eta = \eta_{\mathrm{her}}\eta_{\mathrm{gc}}\eta_{\mathrm{ch}}\eta_{\mathrm{det}}$ represents all losses excluding the transmission in the interferometer. This includes the heralding efficiency η_{her} of the sources, the chip-to-fiber collection efficiency of the grating couplers η_{gc}, the transmittivity η_{ch} of the channel to the detectors, and the detection efficiency η_{det}. The factor $\eta_{\mathrm{u}}^{\mathrm{m}}$ represents the total losses in the interferometer, where η_{u} represents the losses in each coupling operation (e.g. evanescently coupled waveguides in a universal scheme [26, 27]). The number of single operations each photon undergoes in the m mode interferometer is assumed to be m, as in the universal scheme proposed in Ref. [27]. Because in SBS only the signal photons undergo the interference, in Supplementary Equation 30 the exponent for this term is $n/2$ instead of n. We consider here experiments with off-chip detectors using state-of-the-art silicon grating couplers with $\eta_{\mathrm{gc}} = 0.89$, which are being already employed in sampling experiments with silicon quantum photonics [28, 29]. We assume one source in each input mode of the interferometer ($m = k$), as is standard

Event rates (Hz)	Number of photons				
Number of sources	4	8	12	16	20
4	2×10^4	2×10^{-1}	-	-	-
8	6×10^4	9×10^0	2×10^{-4}	-	-
16	2×10^5	2×10^2	4×10^{-2}	-	-
32	5×10^5	2×10^3	2×10^0	1×10^{-3}	-
64	7×10^5	9×10^3	5×10^1	1×10^{-1}	2×10^{-4}
128	3×10^5	1×10^4	2×10^2	2×10^0	1×10^{-2}

Supplementary Table 2. Simulated rates for NISQ scattershot boson sampling implementations with current silicon quantum photonics technology using our sources.

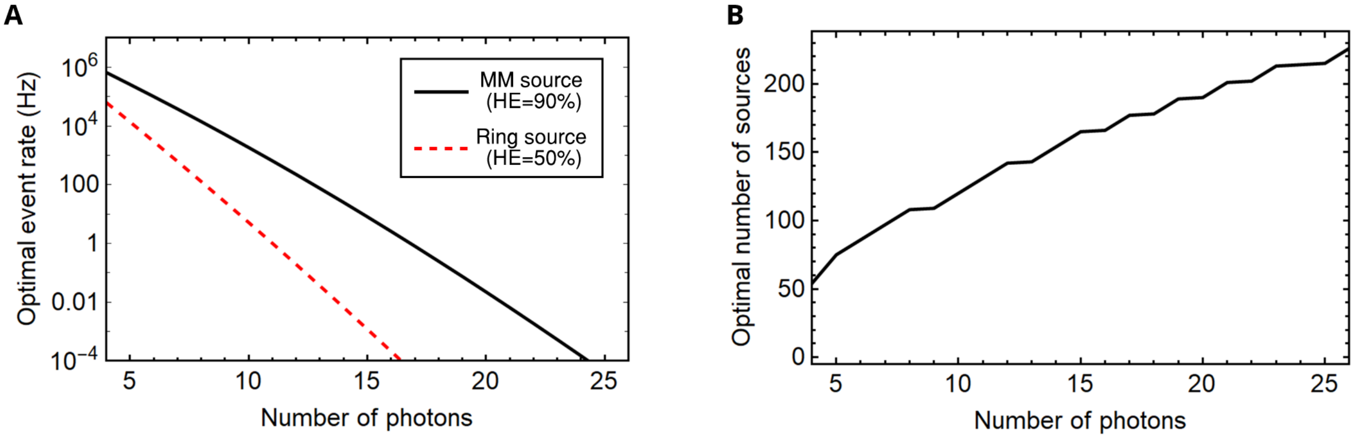

Supplementary Figure 9. **a)** Estimated optimal rates for SBS experiments with different numbers of photons using our multi-modal sources (black solid line, $\eta_{\text{he}} = 0.9$), compared to the rates considering standard rings (red dashed line, $\eta_{\text{he}} = 0.5$). **b)** Number of sources associated to the optimal rates for different photon numbers. The optimal configuration is the same for both multi-modal and standard ring sources.

for SBS. For all the other parameters we consider the values characterised in our device: $R_0 = 50$ MHz, $\eta_{\text{ch}} = 0.8$, $\eta_{\text{det}} = 0.8$, $\eta_{\text{u}} = 0.995$, and $\eta_{\text{her}} = 0.9$ for our multi-mode sources. For the squeezing we use $\tanh(\xi)^2 = 0.03$, which is approximately what we used in our heralded HOM experiment.

In Supplementary Table 2 we report the estimated rates for events with different photon numbers and numbers of sources. The rates indicate that with approximately $k = 100$ mode devices, possible with current technologies, experiments with ≥ 20 photons are possible. Experiments at this scale are expected to enter in a computationally interesting regime where they can compete with classical supercomputers in solving sampling problems [30]. Note that while scattershot-type rate enhancement can be initially observed when increasing the number of sources $k = m$, when it becomes too large increasing the modes in the circuit becomes detrimental as losses in the interferometer become dominant. The optimal rates are thus obtained at a trade-off between these two effects, and depend on the number of photons and losses in the circuit. The optimal rates, using the same parameters used to obtain Supplementary Table 2, are reported for different numbers of photons in Supplementary Figure 9a. The associated optimal number of sources for different numbers of photons are shown in Supplementary Figure 9b. For comparison, we also report in Supplementary Figure 9a the equivalent optimal rates in the case ring resonator sources with a heralding efficiency of $\eta_{\text{her}} = 0.5$ (which is the heralding value for critically coupled rings, where the source brightness is maximal [12]) are used, which represent the state-of-the-art for silicon-based integrated photon sources. In this comparison, with our high-heralding efficiency photon sources the rates are improved by several orders of magnitude when increasing the number of photons. For example, it can be observed that 20 photons events can be achieved at Hz rates using the optimal number of 130 multi-modal sources, while experiments at this size could not be achievable using standard ring sources with the current silicon photonics technology. We also remark that these rates can be dramatically further enhanced with near-term integrated quantum photonic technologies, e.g. the large-scale integration of high-efficiency detectors [31].

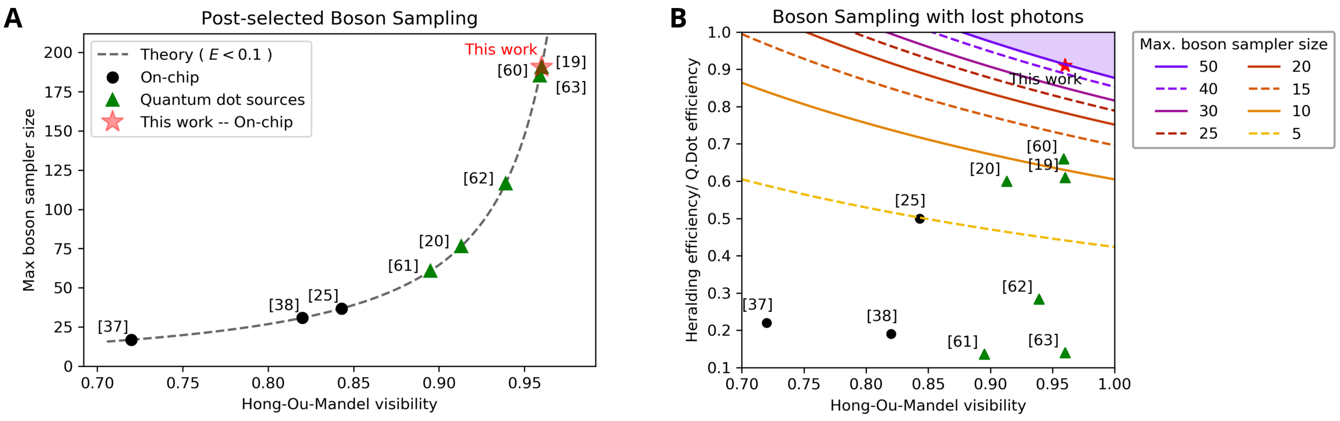

Supplementary Figure 10. Estimated maximum boson sampler size thresholds k calculated via Supplementary Equation 31 for standard boson-sampling with post-selection **(a)** and for boson sampling with lost photons **(b)**. The red star represents values for our source, black circles are for on-chip sources, and green triangles are for quantum dot emitters. References for each value of raw HOM visiblity and efficiency are shown next to the associated data point. Solid and dashed lines are theoretical values calculated via Supplementary Equation 31 considering classical error bounds of 10%, as in Ref. [38].

b. Quantum-to-classical threshold estimations

Although increasing the number of photons is crucial to reach computationally interesting regimes with NISQ photonic sampling machines, the quality of the photons is also a key parameter to take into account. In fact, for a constant noise per photon (e.g. due to non-unit photon purity or indistinguishability), errors build up rapidly when increasing the photon number. If the photon number is too large, errors become large enough to enable efficient classical simulations of the experiment. In particular, recent works have developed tools to quantify a photon number threshold level k, that depends only on the quality of the single photons and circuits used, such that any experiment with $n > k$ photons can be considered equivalent to an ideal experiment with k ideal photons (classically hard to simulate) and $n-k$ distinguishable photons (classically easy to simulate) [38]. In other terms, the threshold k provides an upper bound to the computational complexity of boson sampling experiments achievable with photons and circuits of a given quality. More in details, the bound k relates to the error bound E of the classical approximation as [38]:

$$E < \sqrt{\frac{\alpha^{(k+1)}}{1-\alpha}}. \tag{31}$$

The parameter α represents a quality factor given by

$$\alpha = \eta \mathcal{V} \tag{32}$$

where $\mathcal{V}$ the Hong-Ou-Mandel interference visibility (which includes the imperfection from photon indistinguishability and non-unit purity), and $\eta = n_{\mathrm{o}}/n_{\mathrm{i}}$ represents the fraction of photons detected at the output n_{o} and the number of input photons n_{i}. In standard boson sampling, post-selection is used to impose $n_{\mathrm{o}} = n_{\mathrm{i}}$, that is $\eta = 1$. Note however that, when inefficiencies are present (e.g. limited heralding efficiency of photon pair sources, non-deterministic generation from single photon emitters, or losses in the circuit), such post-selection procedure can decrease the event rates significantly and limits the scaling to high photon numbers. However, a different approach is to avoid post-selection and consider photon loss as part of the sampling problem [39]. In this case the sampling is performed in both the number of output photons observed and the detected configuration from the output distribution associated to that photon number [38]. In this case $n_{\mathrm{o}} = \eta n_{\mathrm{i}}$ represents the average photon number at the output, with now $\eta < 1$.

Thresholds values estimated via Supplementary Equation 31 are reported in Supplementary Figure 10a for the standard boson sampling case, and in Supplementary Figure 10b for boson sampling with lost photons (considering classical error bounds of 10% [38]). In Supplementary Figure 10b we take the same approach as Ref. [40] and consider η as the probability to obtain a photon from a source given that we expect to have one. For spontaneous photon sources this corresponds to the heralding efficiency, while for single photon emitters it corresponds to system efficiency. Note that, while this assumption is useful to compare the capability of different sources to support experiments with

high computational complexity, the value reported should only be considered as upper bounds to the threshold, as additional losses in the circuits are neglected. The thresholds associated to our source are shown as red stars. For comparison, we also show the thresholds associated to various sources reported in the literature for two of the main current approaches to generate photons for NISQ architectures: quantum dot single-photon emitters (green triangles) and integrated spontaneous photon sources for on-chip experiments (black circles). In the standard boson sampling case (Supplementary Figure 10a) with post-selection, our source improves dramatically the maximum boson sampler size k when compared to previous on-chip photon sources, and obtains comparable results with state-of-the-art quantum dot emitters. However, when considering the boson sampling with lost photons approach (Supplementary Figure 10b) our source provides a significant advantage also respect to quantum dots.

Supplementary Note 5: Measurements of squeezing from two-photon correlations using threshold detectors

We describe here in details the methods used to calculate the two-mode squeezing (TMS) parameter of the source emission using two-mode second order correlation measurements in our experiment with the set-up schematised in Supplementary Figure 1d). The method we use is very similar to the one reported in Ref. [41], with the only difference that non-number resolving detectors are considered here to represents the actual experimental set-up. The two results coincide in the low-squeezing regime, where multi-photon detection terms become negligible.

Given a pure single mode TMS state, which is a valid approximation for the highly spectrally pure source emission in our experiment, the probability of detecting n_s (n_i) single photons in the signal (idler) mode in the loss-less case is given by

$$p_{\mathrm{TMS}}(n_\mathrm{s}, n_\mathrm{i}) = \frac{\left(\tanh(\xi)^{2n_\mathrm{s}}\right)}{\cosh^2(\xi)}\delta_{n_\mathrm{s},n_\mathrm{i}} = p_0(n_\mathrm{s})\delta_{n_\mathrm{s},n_\mathrm{i}}, \tag{33}$$

with $p_0(n) = \tanh(\xi)^{2n}/\cosh^2(\xi)$. The Kronecker delta here represents the fact that in an ideal TMS the number of signal and idler photons are perfectly correlated. In presence of losses, where the transmission in the signal and idler modes are given by η_s and η_i respectively, the detection probability is given by

$$p(n_\mathrm{s}, n_\mathrm{i}) = \sum_{n \geq \max\{n_\mathrm{s}, n_\mathrm{i}\}} p_0(n)\eta_s^{n_\mathrm{s}}(1-\eta_\mathrm{s})^{n-n_\mathrm{s}}\eta_\mathrm{s}^{n_\mathrm{i}}(1-\eta_\mathrm{s})^{n-n_\mathrm{i}}, \tag{34}$$

which takes into account all possible ways of generating a larger number of n signal and idler photons, and then losing exactly $n - n_\mathrm{s}$ signal photons and $n - n_\mathrm{i}$ idler photons. We will from now on consider for simplicity uniform losses in the signal and idler modes ($\eta_\mathrm{s} = \eta_\mathrm{i} = \eta$), although all results can be generalised to the non-uniform case straightforwardly. In this approximation we obtain

$$\begin{aligned} p(n_\mathrm{s}, n_\mathrm{i}) &= \left(\frac{\eta}{1-\eta}\right)^{n_\mathrm{s}+n_\mathrm{i}} \sum_{n \geq \max\{n_\mathrm{s}, n_\mathrm{i}\}} p_0(n)(1-\eta)^{-2n} \\ &= A(\xi, \eta)\left(\frac{\eta}{1-\eta}\right)^{n_\mathrm{s}+n_\mathrm{i}} \sum_{n \geq \max\{n_\mathrm{s}, n_\mathrm{i}\}} \left[\tanh^2(\xi)(1-\eta)^2\right]^n \\ &= B(\xi, \eta)\left(\frac{\eta}{1-\eta}\right)^{n_\mathrm{s}+n_\mathrm{i}} \left[\tanh(\xi)(1-\eta)\right]^{2\max\{n_\mathrm{s}, n_\mathrm{i}\}}, \end{aligned} \tag{35}$$

where $A(\xi, \eta)$ and $B(\xi, \eta)$ are normalisation constant and we have used the geometric series formula. Now, to determine the constant $B(\xi, \eta)$ we impose the normalisation condition $1 = \sum_{n_\mathrm{s}, n_\mathrm{i}=0}^{\infty} p(n_\mathrm{s}, n_\mathrm{i})$ and rewrite

$$1 = \sum_{n_\mathrm{s}, n_\mathrm{i}=0}^{\infty} p(n_\mathrm{s}, n_\mathrm{i}) = 2\sum_{n_\mathrm{i}=0}^{\infty}\sum_{n_\mathrm{s} \geq n_\mathrm{i}}^{\infty} p(n_\mathrm{s}, n_\mathrm{i}) - \sum_{n=0}^{\infty} p(n, n). \tag{36}$$

The two sums on the right now simply represent cascaded geometrical series, and result in

$$1 = \frac{B(\xi, \eta)}{\zeta(a)(1-\eta^2\tanh^2\xi)}, \tag{37}$$

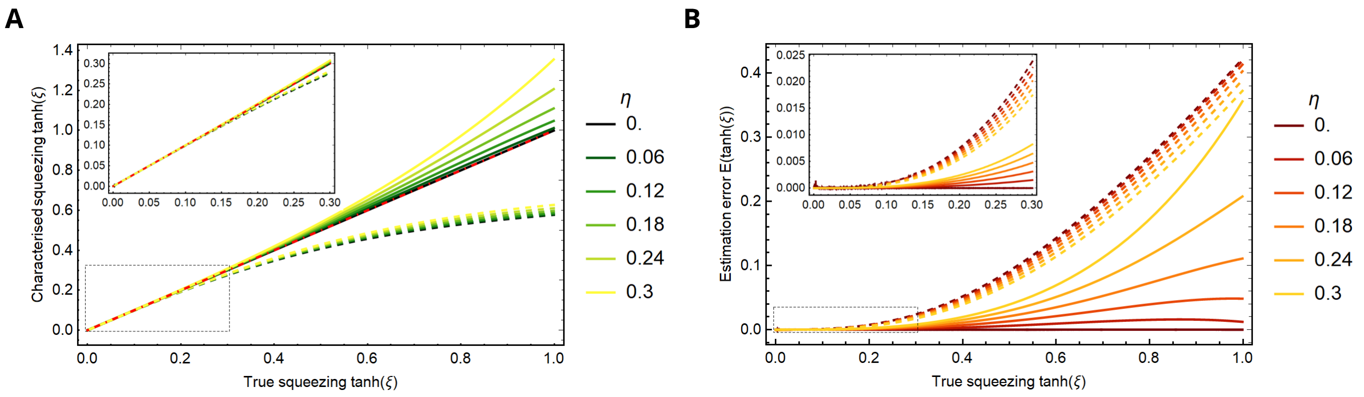

Supplementary Figure 11. a) Estimation of the squeezing parameter ξ via second-order correlations in presence of losses and threshold detectors. Dashed lines correspond to estimating $\tanh^2(\xi)$ directly as the ratio R, while solid lines include the expansion in Supplementary Equation (42). The red dash-dotted line represents the true squeezing value. b) Error in the estimation with the two different approaches for various squeezing values and losses.

with $a = \eta(1-\eta)\tanh^2(\xi)$ and $\zeta(x) = (1-x)/(1+x)$, giving

$$B(\xi,\eta) = \zeta(a)(1-\eta^2\tanh^2\xi). \tag{38}$$

The function $p(n_\mathrm{s}, n_\mathrm{i})$ thus indicate the general probability of detecting n_i idler photons and n_s signal photons from a single TMS in presence of lossy channels with transmittivity η.

We now consider performing second-order correlation measurements on this state, with the additional complexity of using non-number-resolving (i.e. threshold) detectors (as is the case in our experiments, see Supplementary Figure 1d). Using threshold detectors, to two detectors will produce a coincidence event whenever at least one photon arrives to each one of them. We also consider operating the TMS source in a pulsed pumping regime. Correlation measurements at time delay $\Delta t = 0$ will thus represents events where photons emitted in the same TMS are detected, while a time delay $\Delta t > 0$ indicates events where photons belonging to different TMS (emitted with pulses at different times) are detected. At time $\Delta t = 0$, this occurs whenever $n_\mathrm{s} \geq 1$ signal photons and $n_\mathrm{i} \geq 1$ idler photons from a single TMS arrive at the detectors, which happens with probability

$$p_\mathrm{coinc}(\Delta t = 0) = p(n_\mathrm{s} \geq 1, n_i \geq 1) = 1 - p(0,0) - p(0,1) - p(1,0) = 1 - B(1+2a). \tag{39}$$

Coincidence events at times $\Delta t > 0$ are instead obtained if $n_\mathrm{s} \geq 1$ signal photons arrive from a TMS emitted at time 0, and $n_\mathrm{i} \geq 1$ idler photons arrive from a second TMS emitted at time Δt. The probability of this event to happen is

$$\begin{aligned} p_\mathrm{coinc}(\Delta t > 0) &= p(n_\mathrm{s} \geq 1) p(n_\mathrm{i} \geq 1) \\ &= \left(\sum_{n_\mathrm{i}=0}^{\infty} p(n_\mathrm{s} \geq 1, n_\mathrm{i})\right)\left(\sum_{n_\mathrm{s}=0}^{\infty} p(n_\mathrm{s}, n_\mathrm{i} \geq 1)\right) \\ &= \left(1 - \frac{B}{1-a}\right)^2. \end{aligned} \tag{40}$$

Now, the quantity we consider is the ratio between the coincidences measured at $\Delta t > 0$ and those measured at $\Delta t = 0$:

$$R = \frac{p_\mathrm{coinc}(\Delta t > 0)}{p_\mathrm{coinc}(\Delta t = 0)}. \tag{41}$$

It is now useful to consider the approximation $\eta \tanh^4 \xi \approx 0$, which is typical in conditions where the squeezing is low and losses in the detection channels are (or can be made, if needed) significant, as in our experimental set-up. Under this approximation, we can expand Supplementary Equation (41) as:

$$R = \frac{\tanh^2 \xi}{1 + 2\tanh^2 \xi} + \mathcal{O}\left(\eta \tanh^4 \xi\right), \tag{42}$$

which finally provides an estimation of the squeezing parameter via:

$$\tanh^2 \xi \simeq \frac{R}{1 - 2R}. \tag{43}$$

Note that for $\tanh^2 \xi \to 0$ this equation converges to $\tanh^2 \xi \simeq R$ as in Ref. [41], as expected (number-resolving and threshold detection give same results in a low-squeezing regime). In Supplementary Figure 11 we compare the squeezing estimation via the standard $\tanh^2 \xi \simeq R$ formula or via following Supplementary Equation (43).

Supplementary Note 6: Measurements of source indistinguishability

a. *Measurement of indistinguishability via the reversed Hong-Ou-Mandel effect*

We now directly link the overlap between the JSAs from two different sources to the visibility of revHOM experiments. A similar analysis, using a simplified model approximation, was previously reported in [42]. Here, we formulate the analysis using a full description of the biphoton spectral state. In the revHOM effect, we inject a superposition state

$$|\psi\rangle = \frac{N}{\sqrt{2}}(|\psi\rangle_1 + e^{i2\phi} |\psi\rangle_2) \tag{44}$$

into a beam-splitter [43], where $|\psi\rangle_1$ and $|\psi\rangle_2$ are (unnormalised) states associated to the first and second source of photons injected into the two input modes of the beam-splitter. In the low-squeezing regime, we can write these states via biphoton wavefunctions:

$$|\psi\rangle_1 = \int d\omega_i d\omega_s \; \psi_1(\omega_i, \omega_s)\hat{a}_1^\dagger(\omega_i)\hat{b}_1^\dagger(\omega_s)|0\rangle, \tag{45}$$

$$|\psi\rangle_2 = \int d\omega_i d\omega_s \; \psi_2(\omega_i, \omega_s)\hat{a}_2^\dagger(\omega_i)\hat{b}_2^\dagger(\omega_s)|0\rangle. \tag{46}$$

Here, $\psi_1(\omega_i, \omega_s)$ and $\psi_2(\omega_i, \omega_s)$ are the two (unnormalised) JSAs for the biphoton state on each mode. The factor N is a normalisation constant, which, imposing the normalisation of $|\psi\rangle$, is given by

$$N^2 = \frac{2}{\int d\omega_i d\omega_s |\psi_1(\omega_i, \omega_s)|^2 + \int d\omega_i d\omega_s |\psi_2(\omega_i, \omega_s)|^2} \tag{47}$$

The action of the beam-splitter (which, for simplicity, is supposed to be frequency-independent) can be written as the transformation of the bosonic operators

$$\hat{a}_1^\dagger(\omega) \mapsto \left[\hat{a}_1^\dagger(\omega) + i\hat{a}_2^\dagger(\omega)\right]/\sqrt{2}, \qquad \hat{a}_2^\dagger(\omega) \mapsto \left[i\hat{a}_1^\dagger(\omega) + \hat{a}_2^\dagger(\omega)\right]/\sqrt{2}, \tag{48}$$

$$\hat{b}_1^\dagger(\omega) \mapsto \left[\hat{b}_1^\dagger(\omega) + i\hat{b}_2^\dagger(\omega)\right]/\sqrt{2}, \qquad \hat{b}_2^\dagger(\omega) \mapsto \left[i\hat{b}_1^\dagger(\omega) + \hat{b}_2^\dagger(\omega)\right]/\sqrt{2}. \tag{49}$$

The output state is thus given by

$$|\psi\rangle \mapsto \quad \frac{N}{2\sqrt{2}} \int d\omega_i d\omega_s \; \psi_1(\omega_i, \omega_s)\left[\left(\hat{a}_1^\dagger(\omega_i) + i\hat{a}_2^\dagger(\omega_i)\right)\left(\hat{b}_1^\dagger(\omega_s) + i\hat{b}_2^\dagger(\omega_s)\right)\right]|0\rangle \tag{50}$$

$$+e^{i2\phi}\psi_2(\omega_i, \omega_s)\left[\left(i\hat{a}_1^\dagger(\omega_i) + \hat{a}_2^\dagger(\omega_i)\right)\left(i\hat{b}_1^\dagger(\omega_s) + \hat{b}_2^\dagger(\omega_s)\right)\right]|0\rangle \tag{51}$$

$$= \quad \frac{1}{\sqrt{2}}(|\text{bunch}\rangle + |\text{split}\rangle) \tag{52}$$

where

$$|\text{bunch}\rangle = \frac{N}{2}\int d\omega_{\rm i} d\omega_{\rm s} \left[\hat{a}_2^\dagger(\omega_{\rm i})\hat{b}_2^\dagger(\omega_{\rm s}) - \hat{a}_1^\dagger(\omega_{\rm i})\hat{b}_1^\dagger(\omega_{\rm s})\right]\left(e^{i2\phi}\psi_2(\omega_{\rm i},\omega_{\rm s}) - \psi_1(\omega_{\rm i},\omega_{\rm s})\right)|0\rangle \tag{53}$$

$$|\text{split}\rangle = \frac{iN}{2}\int d\omega_{\rm i} d\omega_{\rm s} \left[\hat{a}_1^\dagger(\omega_i)\hat{b}_2^\dagger(\omega_{\rm s}) + \hat{a}_2^\dagger(\omega_{\rm i})\hat{b}_1^\dagger(\omega_{\rm s})\right]\left(e^{i2\phi}\psi_2(\omega_{\rm i},\omega_{\rm s}) + \psi_1(\omega_{\rm i},\omega_{\rm s})\right)|0\rangle\,. \tag{54}$$

Here, $|\text{bunch}\rangle$ represents the cases where the output photons bunch in the same output mode, while the the term $|\text{split}\rangle$ represents the cases where photons emerge from different modes. If our measurements are frequency insensitive (i.e. the detectors do not resolve different wavelengths), then the single-photon detection projective operators on the two different output modes are given by:

$$\hat{P}_{1,\rm i} = \int d\omega_1\ \hat{a}_1^\dagger(\omega_1)|0\rangle\langle 0|\hat{a}_1(\omega_1), \qquad \hat{P}_{2,\rm i} = \int d\omega_2\ \hat{a}_2^\dagger(\omega_2)|0\rangle\langle 0|\hat{a}_2(\omega_2), \tag{55}$$

$$\hat{P}_{1,\rm s} = \int d\omega_1\ \hat{b}_1^\dagger(\omega_1)|0\rangle\langle 0|\hat{b}_1(\omega_1), \qquad \hat{P}_{2,\rm s} = \int d\omega_2\ \hat{b}_2^\dagger(\omega_2)|0\rangle\langle 0|\hat{b}_2(\omega_2), \tag{56}$$

and the projective operator for a coincidence measurement, e.g. simultaneous detection on modes 1 for the idler and 2 for the signal, is given by

$$\hat{P}_{1,\rm i}\otimes\hat{P}_{2,\rm s} = \int d\omega_1 d\omega_2\ \hat{a}_1^\dagger(\omega_1)\hat{b}_2^\dagger(\omega_2)|0\rangle\langle 0|\hat{a}_1(\omega_1)\hat{b}_2(\omega_2). \tag{57}$$

The coincidence probability is thus given by the expectation value $p_{\rm coinc} = \langle\psi|\hat{P}_1\otimes\hat{P}_2|\psi\rangle$. Now, the term $|\text{bunch}\rangle$, which only contains terms $\hat{a}_1^\dagger\hat{b}_1^\dagger$ or $\hat{a}_2^\dagger\hat{b}_2^\dagger$, necessarily gives zero contribution to this expectation value. We thus have

$$p_{\rm coinc} = \langle\psi|\hat{P}_1\otimes\hat{P}_2|\psi\rangle\,, \tag{58}$$

$$= \frac{1}{4} + \frac{N^2}{4}\,\text{Re}\left[e^{i2\phi}\int d\omega_1 d\omega_2\ \psi_1^*(\omega_1,\omega_2)\psi_2(\omega_1,\omega_2)\right], \tag{59}$$

Defining $\theta_0 = \arg\int d\omega_1 d\omega_2 \psi_2^*(\omega_1,\omega_2)\psi_1(\omega_1,\omega_2)$, we finally arrive at

$$p_{\rm coinc} = \frac{1}{4} + \frac{N^2}{4}\cos(2\phi+\theta_0)\left|\int d\omega_1 d\omega_2\ \psi_1^*(\omega_1,\omega_2)\psi_2(\omega_1,\omega_2)\right| \tag{60}$$

which represents the revHOM fringe. The visibility of this fringe is given by

$$\mathcal{V}_{\rm revHOM} = N^2\left|\int d\omega_1 d\omega_2\ \psi_2^*(\omega_1,\omega_2)\psi_1(\omega_1,\omega_2)\right| \equiv \mathcal{I} \tag{61}$$

which is the normalised overlap between the two JSAs, i.e. the indistinguishability $\mathcal{I}$ between the two sources. Note that, on the other hand, the revHOM visibility does not depend on the separability of either JSA, in contrast to the heralded-HOM effect. It thus allows us to characterise sources indistinguishability independently from the heralded photon purities.

b. Effects of distinguishability on the generated entanglement and indistinguishability estimation via two-qubit tomography

We investigate how the source distinguishability affects the two-qubit entanglement generated in the scheme in Fig. 2b (and equivalently in, for example, Refs. [10, 42]), and how this effect can be used to characterise the indistinguishability via reconstructing the two-qubit state encoded in the photons. As in the previous section, in the low-squeezing approximation we write the input state as the superposition $|\psi\rangle = N(|\psi\rangle_1 + |\psi\rangle_2)/\sqrt{2}$, with N the normalisation constant in Supplementary Equation (47) and $|\psi\rangle_1$ and $|\psi\rangle_2$ the two biphoton states in Supplementary Equation (45) and Supplementary Equation (46). The logical encoding of a qubit on each photon proceeds as follows [10]: we say that the qubit encoded in the signal (idler) photon is in the state $|0\rangle^{(L)}$ if the photon is in the spatial mode associated to $\hat{a}_1$ ($\hat{b}_1$), while $|1\rangle^{(L)}$ if it is in the spatial mode associated to $\hat{a}_2$ ($\hat{b}_2$). We use here the superscript L to differentiate logical states of qubits to Fock photonic states. This encoding depends only on the spatial properties of the photons, while it is independent from the frequencies. In the experimental scenario considered

here, also the single-photon measurements are not frequency-resolving, and we can thus write down the qubit projectors in the computational (Pauli Z) basis via the projectors as in Supplementary Equation (55) and Supplementary Equation (56):

$$|0\rangle\langle 0|_{\mathrm{i}}^{(\mathrm{L})} = \int d\omega_1\ \hat{a}_1^\dagger(\omega_1)\,|0\rangle\langle 0|\,\hat{a}_1(\omega_1), \qquad |1\rangle\langle 1|_{\mathrm{i}}^{(\mathrm{L})} = \int d\omega_2\ \hat{a}_2^\dagger(\omega_2)\,|0\rangle\langle 0|\,\hat{a}_2(\omega_2), \tag{62}$$

$$|0\rangle\langle 0|_{\mathrm{s}}^{(\mathrm{L})} = \int d\omega_1\ \hat{b}_1^\dagger(\omega_1)\,|0\rangle\langle 0|\,\hat{b}_1(\omega_1), \qquad |1\rangle\langle 1|_{\mathrm{s}}^{(\mathrm{L})} = \int d\omega_2\ \hat{b}_2^\dagger(\omega_2)\,|0\rangle\langle 0|\,\hat{b}_2(\omega_2). \tag{63}$$

The expectation value for two-qubits observables on the photon-encoded logical state can thus me obtained via:

$$p(|00\rangle) = \langle\psi|\left[|0\rangle\langle 0|_{\mathrm{i}}^{(\mathrm{L})} \otimes |0\rangle\langle 0|_{\mathrm{s}}^{(\mathrm{L})}\right]|\psi\rangle = \int d\omega_{\mathrm{i}} d\omega_{\mathrm{s}}\, |\psi_1(\omega_{\mathrm{i}},\omega_{\mathrm{s}})|^2\,, \tag{64}$$

$$p(|01\rangle) = \langle\psi|\left[|0\rangle\langle 0|_{\mathrm{i}}^{(\mathrm{L})} \otimes |1\rangle\langle 1|_{\mathrm{s}}^{(\mathrm{L})}\right]|\psi\rangle = 0, \tag{65}$$

$$p(|10\rangle) = \langle\psi|\left[|1\rangle\langle 1|_{\mathrm{i}}^{(\mathrm{L})} \otimes |0\rangle\langle 0|_{\mathrm{s}}^{(\mathrm{L})}\right]|\psi\rangle = 0, \tag{66}$$

$$p(|11\rangle) = \langle\psi|\left[|1\rangle\langle 1|_{\mathrm{i}}^{(\mathrm{L})} \otimes |1\rangle\langle 1|_{\mathrm{s}}^{(\mathrm{L})}\right]|\psi\rangle = \int d\omega_{\mathrm{i}} d\omega_{\mathrm{s}}\, |\psi_2(\omega_{\mathrm{i}},\omega_{\mathrm{s}})|^2\,. \tag{67}$$

Using local transformations as those in Supplementary Equation (48) and Supplementary Equation (49), one can also write down the logical projective operators in the Pauli X and Y bases:

$$|+\rangle\langle +|_{\mathrm{i}}^{(\mathrm{L})} = \frac{1}{2}\int d\omega \left[\hat{a}_1^\dagger(\omega)\,|0\rangle\langle 0|\,\hat{a}_1(\omega) + \hat{a}_1^\dagger(\omega)\,|0\rangle\langle 0|\,\hat{a}_2(\omega) + \hat{a}_2^\dagger(\omega)\,|0\rangle\langle 0|\,\hat{a}_1(\omega) + \hat{a}_2^\dagger(\omega)\,|0\rangle\langle 0|\,\hat{a}_2(\omega)\right], \tag{68}$$

$$|-\rangle\langle -|_{\mathrm{i}}^{(\mathrm{L})} = \frac{1}{2}\int d\omega \left[\hat{a}_1^\dagger(\omega)\,|0\rangle\langle 0|\,\hat{a}_1(\omega) - \hat{a}_1^\dagger(\omega)\,|0\rangle\langle 0|\,\hat{a}_2(\omega) - \hat{a}_2^\dagger(\omega)\,|0\rangle\langle 0|\,\hat{a}_1(\omega) + \hat{a}_2^\dagger(\omega)\,|0\rangle\langle 0|\,\hat{a}_2(\omega)\right], \tag{69}$$

$$|{+\mathrm{i}}\rangle\langle {+\mathrm{i}}|_{\mathrm{i}}^{(\mathrm{L})} = \frac{1}{2}\int d\omega \left[\hat{a}_1^\dagger(\omega)\,|0\rangle\langle 0|\,\hat{a}_1(\omega) + \mathrm{i}\hat{a}_1^\dagger(\omega)\,|0\rangle\langle 0|\,\hat{a}_2(\omega) - \mathrm{i}\hat{a}_2^\dagger(\omega)\,|0\rangle\langle 0|\,\hat{a}_1(\omega) + \hat{a}_2^\dagger(\omega)\,|0\rangle\langle 0|\,\hat{a}_2(\omega)\right], \tag{70}$$

$$|{-\mathrm{i}}\rangle\langle {-\mathrm{i}}|_{\mathrm{i}}^{(\mathrm{L})} = \frac{1}{2}\int d\omega \left[\hat{a}_1^\dagger(\omega)\,|0\rangle\langle 0|\,\hat{a}_1(\omega) - \mathrm{i}\hat{a}_1^\dagger(\omega)\,|0\rangle\langle 0|\,\hat{a}_2(\omega) + \mathrm{i}\hat{a}_2^\dagger(\omega)\,|0\rangle\langle 0|\,\hat{a}_1(\omega) + \hat{a}_2^\dagger(\omega)\,|0\rangle\langle 0|\,\hat{a}_2(\omega)\right], \tag{71}$$

and similarly for the qubit encoded in the signal photon. Using these projectors we can calculate the expectation values for the Pauli observables

$$\langle XX\rangle = -\langle YY\rangle = N^2\,\mathrm{Re}\left[\int d\omega_1 d\omega_2\ \psi_2^*(\omega_1,\omega_2)\psi_1(\omega_1,\omega_2)\right], \tag{72}$$

where a dependence on the two JSAs overlap is manifested. To quantify how the generated entanglement between the two qubits is related to the indistinguishability, a convenient quantity to consider is the fidelity $\mathcal{F}_{\Phi_+} = \langle\Phi_+|\,\rho\,|\Phi_+\rangle$ of the two-qubit state ρ with the maximally entangled Bell state $|\Phi_+\rangle = (|00\rangle + |11\rangle)/\sqrt{2}$. This fidelity can be calculated as [44]

$$\mathcal{F}_{\Phi_+} = \frac{p(|00\rangle) + p(|11\rangle) + \frac{\langle XX\rangle - \langle YY\rangle}{2}}{2} = \frac{\rho_{00} + \rho_{11} + N^2\,\mathrm{Re}\left[\int d\omega_1 d\omega_2\ \psi_2^*(\omega_1,\omega_2)\psi_1(\omega_1,\omega_2)\right]}{2}, \tag{73}$$

which, given the reconstructed density matrix ρ, allows us to obtain a lower bound on the source indistinguishability via

$$\mathcal{I} \equiv N^2\left|\int d\omega_1 d\omega_2\ \psi_2^*(\omega_1,\omega_2)\psi_1(\omega_1,\omega_2)\right| \geq N^2\,\mathrm{Re}\left[\int d\omega_1 d\omega_2\ \psi_2^*(\omega_1,\omega_2)\psi_1(\omega_1,\omega_2)\right] = 2\mathcal{F}_{\Phi_+} - \rho_{00} - \rho_{11}. \tag{74}$$

The bound becomes exact if we consider the maximum fidelity with maximally entangled states of the form $|\Phi(\varphi)\rangle = (|00\rangle + \mathrm{e}^{\mathrm{i}\varphi}\,|11\rangle)/\sqrt{2}$, that is $\mathcal{F}_{\max} = \max_\varphi \langle\Phi(\varphi)|\,\rho\,|\Phi(\varphi)\rangle$. This fidelity quantifies how close the state is to being maximally entangled, thus expressing the entanglement in the two-qubit state. Once the density matrix ρ is reconstructed via quantum state tomography, $\mathcal{F}_{\max}$ can be calculated via a simple numerical maximisation. In this case we can estimate the source indistinguishability via:

$$\mathcal{I} = 2\mathcal{F}_{\max} - \rho_{00} - \rho_{11}. \tag{75}$$

This equation was used in the main text to estimate the source indistinguishability from the density matrix reconstructed in Supplementary Equation 2e.

Supplementary References

[1] Stefano Signorini, Mattia Mancinelli, Massimo Borghi, Martino Bernard, Mher Ghulinyan, Georg Pucker, and Lorenzo Pavesi, "Intermodal four-wave mixing in silicon waveguides," Photon. Res. **6**, 805–814 (2018).

[2] Bin Fang, Offir Cohen, Jamy B Moreno, and Virginia O Lorenz, "State engineering of photon pairs produced through dual-pump spontaneous four-wave mixing," Opt. Express **21**, 2707–2717 (2013).

[3] Z Vernon, M Menotti, CC Tison, JA Steidle, ML Fanto, PM Thomas, SF Preble, AM Smith, PM Alsing, M Liscidini, *et al.*, "Truly unentangled photon pairs without spectral filtering," Opt. Lett. **42**, 3638–3641 (2017).

[4] K Garay-Palmett, HJ McGuinness, Offir Cohen, JS Lundeen, R Rangel-Rojo, AB Uren, MG Raymer, CJ McKinstrie, S Radic, and IA Walmsley, "Photon pair-state preparation with tailored spectral properties by spontaneous four-wave mixing in photonic-crystal fiber," Opt. Express **15**, 14870–14886 (2007).

[5] Lijian Zhang, Christoph Söller, Offir Cohen, Brian J Smith, and Ian A Walmsley, "Heralded generation of single photons in pure quantum states," Journal of Modern Optics **59**, 1525–1537 (2012).

[6] Matteo Cherchi, Sami Ylinen, Mikko Harjanne, Markku Kapulainen, and Timo Aalto, "Dramatic size reduction of waveguide bends on a micron-scale silicon photonic platform," Opt. Express **21**, 17814–17823 (2013).

[7] "Nanophotonic fdtd simulation software - lumerical fdtd," `https://www.lumerical.com/products/fdtd/`.

[8] Hui Chen and Andrew W Poon, "Low-loss multimode-interference-based crossings for silicon wire waveguides," IEEE photonics technology letters **18**, 2260–2262 (2006).

[9] Hongnan Xu and Yaocheng Shi, "Dual-mode waveguide crossing utilizing taper-assisted multimode-interference couplers," Opt. Lett. **41**, 5381–5384 (2016).

[10] Jianwei Wang, Stefano Paesani, Yunhong Ding, Raffaele Santagati, Paul Skrzypczyk, Alexia Salavrakos, Jordi Tura, Remigiusz Augusiak, Laura Mančinska, Davide Bacco, *et al.*, "Multidimensional quantum entanglement with large-scale integrated optics," Science **360**, 285–291 (2018).

[11] Imad I. Faruque, Gary F. Sinclair, Damien Bonneau, John G. Rarity, and Mark G. Thompson, "On-chip quantum interference with heralded photons from two independent micro-ring resonator sources in silicon photonics," Opt. Express **26**, 20379–20395 (2018).

[12] Z Vernon, M Liscidini, and JE Sipe, "No free lunch: the trade-off between heralding rate and efficiency in microresonator-based heralded single photon sources," Opt. Lett. **41**, 788–791 (2016).

[13] Jesper Bjerge Christensen, Jacob Gade Koefoed, Karsten Rottwitt, and CJ McKinstrie, "Engineering spectrally unentangled photon pairs from nonlinear microring resonators by pump manipulation," Opt. Lett. **43**, 859–862 (2018).

[14] Yingwen Liu, Chao Wu, Xiaowen Gu, Yuechan Kong, Xinxin Yu, Renyou Ge, Xinlun Cai, Xiaogang Qiang, Junjie Wu, Xuejun Yang, and Ping Xu, "High-spectral-purity photon generation from a dual-interferometer-coupled silicon microring," Opt. Lett. **45**, 73–76 (2020).

[15] Jacques Carolan, Uttara Chakraborty, Nicholas C Harris, Mihir Pant, Tom Baehr-Jones, Michael Hochberg, and Dirk Englund, "Scalable feedback control of single photon sources for photonic quantum technologies," Optica **6**, 335–340 (2019).

[16] Justin B Spring, Paolo L Mennea, Benjamin J Metcalf, Peter C Humphreys, James C Gates, Helen L Rogers, Christoph Söller, Brian J Smith, W Steven Kolthammer, Peter GR Smith, *et al.*, "Chip-based array of near-identical, pure, heralded single-photon sources," Optica **4**, 90–96 (2017).

[17] Xiyuan Lu, Steven Rogers, Thomas Gerrits, Wei C. Jiang, Sae Woo Nam, and Qiang Lin, "Heralding single photons from a high-q silicon microdisk," Optica **3**, 1331–1338 (2016).

[18] Lucia Caspani, Chunle Xiong, Benjamin J Eggleton, Daniele Bajoni, Marco Liscidini, Matteo Galli, Roberto Morandotti, and David J Moss, "Integrated sources of photon quantum states based on nonlinear optics," Light: Science & Applications **6**, e17100 (2017).

[19] Damien Bonneau, Gabriel J Mendoza, Jeremy L OBrien, and Mark G Thompson, "Effect of loss on multiplexed single-photon sources," New J. Phys. **17**, 043057 (2015).

[20] A. P. Lund, A. Laing, S. Rahimi-Keshari, T. Rudolph, J. L. O'Brien, and T. C. Ralph, "Boson sampling from a gaussian state," Phys. Rev. Lett. **113**, 100502 (2014).

[21] Michael L Davenport, Songtao Liu, and John E Bowers, "Integrated heterogeneous silicon/iii–v mode-locked lasers," Photonics Research **6**, 468–478 (2018).

[22] Mercedes Gimeno-Segovia, Pete Shadbolt, Dan E. Browne, and Terry Rudolph, "From three-photon greenberger-horne-zeilinger states to ballistic universal quantum computation," Phys. Rev. Lett. **115**, 020502 (2015).

[23] Chris Sparrow, *Quantum Interference in Universal Linear Optical Devices for Quantum Computation and Simulation*, Ph.D. thesis, Department of Physics, Imperial College London (2018).

[24] Austin G. Fowler, Matteo Mariantoni, John M. Martinis, and Andrew N. Cleland, "Surface codes: Towards practical large-scale quantum computation," Phys. Rev. A **86**, 032324 (2012).
[25] Agata M Brańczyk, "Hong-ou-mandel interference," Preprint at https://arxiv.org/abs/1711.00080 (2017).
[26] Michael Reck, Anton Zeilinger, Herbert J. Bernstein, and Philip Bertani, "Experimental realization of any discrete unitary operator," Phys. Rev. Lett. **73**, 58–61 (1994).
[27] William R. Clements, Peter C. Humphreys, Benjamin J. Metcalf, W. Steven Kolthammer, and Ian A. Walmsley, "Optimal design for universal multiport interferometers," Optica **3**, 1460–1465 (2016).
[28] Yunhong Ding, Christophe Peucheret, Haiyan Ou, and Kresten Yvind, "Fully etched apodized grating coupler on the soi platform with- 0.58 db coupling efficiency," Opt. Lett. **39**, 5348–5350 (2014).
[29] Stefano Paesani, Yunhong Ding, Raffaele Santagati, Levon Chakhmakhchyan, Caterina Vigliar, Karsten Rottwitt, Leif K Oxenløwe, Jianwei Wang, Mark G Thompson, and Anthony Laing, "Generation and sampling of quantum states of light in a silicon chip," Nature Phys. **15**, 925–929 (2019).
[30] Alex Neville, Chris Sparrow, Raphaël Clifford, Eric Johnston, Patrick M Birchall, Ashley Montanaro, and Anthony Laing, "Classical boson sampling algorithms with superior performance to near-term experiments," Nature Phys. **13**, 1153–1157 (2017).
[31] Svetlana Khasminskaya, Felix Pyatkov, Karolina Słowik, Simone Ferrari, Oliver Kahl, Vadim Kovalyuk, Patrik Rath, Andreas Vetter, Frank Hennrich, Manfred M Kappes, *et al.*, "Fully integrated quantum photonic circuit with an electrically driven light source," Nature Photon. **10**, 727–732 (2016).
[32] Hui Wang, Yu-Ming He, T-H Chung, Hai Hu, Ying Yu, Si Chen, Xing Ding, M-C Chen, Jian Qin, Xiaoxia Yang, *et al.*, "Towards optimal single-photon sources from polarized microcavities," Nature Photon. **13**, 770–775 (2019).
[33] Xing Ding, Yu He, Z.-C. Duan, Niels Gregersen, M.-C. Chen, S. Unsleber, S. Maier, Christian Schneider, Martin Kamp, Sven Höfling, Chao-Yang Lu, and Jian-Wei Pan, "On-demand single photons with high extraction efficiency and near-unity indistinguishability from a resonantly driven quantum dot in a micropillar," Phys. Rev. Lett. **116**, 020401 (2016).
[34] Hélène Ollivier, Ilse Maillette de Buy Wenniger, Sarah Thomas, Stephen Wein, Guillaume Coppola, Abdelmounaim Harouri, Paul Hilaire, Clément Millet, Aristide Lemaître, Isabelle Sagnes, *et al.*, "Reproducibility of high-performance quantum dot single-photon sources," Preprint at https://arxiv.org/abs/1910.08863 (2019).
[35] Hui Wang, Yu He, Yu-Huai Li, Zu-En Su, Bo Li, He-Liang Huang, Xing Ding, Ming-Cheng Chen, Chang Liu, Jian Qin, *et al.*, "High-efficiency multiphoton boson sampling," Nature Photon. **11**, 361–365 (2017).
[36] Juan C Loredo, Nor A Zakaria, Niccolo Somaschi, Carlos Anton, Lorenzo De Santis, Valerian Giesz, Thomas Grange, Matthew A Broome, Olivier Gazzano, Guillaume Coppola, *et al.*, "Scalable performance in solid-state single-photon sources," Optica **3**, 433–440 (2016).
[37] Niccolo Somaschi, Valerian Giesz, Lorenzo De Santis, JC Loredo, Marcelo P Almeida, Gaston Hornecker, Simone Luca Portalupi, Thomas Grange, Carlos Antón, Justin Demory, *et al.*, "Near-optimal single-photon sources in the solid state," Nature Photon. **10**, 340–345 (2016).
[38] J. J. Renema, A. Menssen, W. R. Clements, G. Triginer, W. S. Kolthammer, and I. A. Walmsley, "Efficient classical algorithm for boson sampling with partially distinguishable photons," Phys. Rev. Lett. **120**, 220502 (2018).
[39] Scott Aaronson and Daniel J. Brod, "Bosonsampling with lost photons," Phys. Rev. A **93**, 012335 (2016).
[40] Reinier van der Meer, Jelmer Jan Renema, Benjamin Brecht, Christine Silberhorn, and Pepijn WH Pinkse, "Optimizing spontaneous parametric down-conversion sources for boson sampling," Preprint at https://arxiv.org/abs/2001.03596 (2020).
[41] Andreas Christ, Kaisa Laiho, Andreas Eckstein, Katiúscia N Cassemiro, and Christine Silberhorn, "Probing multimode squeezing with correlation functions," New J. Phys. **13**, 033027 (2011).
[42] Joshua W Silverstone, Raffaele Santagati, Damien Bonneau, Michael J Strain, Marc Sorel, Jeremy L OBrien, and Mark G Thompson, "Qubit entanglement between ring-resonator photon-pair sources on a silicon chip," Nature Commun. **6**, 7948 (2015).
[43] Joshua W Silverstone, Damien Bonneau, Kazuya Ohira, Nob Suzuki, Haruhiko Yoshida, Norio Iizuka, Mizunori Ezaki, Chandra M Natarajan, Michael G Tanner, Robert H Hadfield, *et al.*, "On-chip quantum interference between silicon photon-pair sources," Nature Photon. **8**, 104–108 (2014).
[44] Otfried Gühne and Géza Tóth, "Entanglement detection," Physics Reports **474**, 1–75 (2009).
